# *CHD8* mutations increase gliogenesis to enlarge brain size in the nonhuman primate

**DOI:** 10.1038/s41421-023-00525-3

**Published:** 2023-03-07

**Authors:** Bang Li, Hui Zhao, Zhuchi Tu, Weili Yang, Rui Han, Lu Wang, Xiaopeng Luo, Mingtian Pan, Xiusheng Chen, Jiawei Zhang, Huijuan Xu, Xiangyu Guo, Sen Yan, Peng Yin, Zhiguang Zhao, Jianrong Liu, Yafeng Luo, Yuefeng Li, Zhengyi Yang, Baogui Zhang, Zhiqiang Tan, Hao Xu, Tianzi Jiang, Yong-hui Jiang, Shihua Li, Yong Q. Zhang, Xiao-Jiang Li

**Affiliations:** 1 Guangdong-Hongkong-Macau Institute of CNS Regeneration, Ministry of Education CNS Regeneration Collaborative Joint Laboratory, Jinan University, Guangzhou, Guangdong China; 2 State Key Laboratory of Molecular Developmental Biology, Institute of Genetics and Developmental Biology, Chinese Academy of Sciences, Beijing, China; 3 Department of Nuclear Medicine and PET/CT-MRI Center, the First Affiliated Hospital of Jinan University & Institute of Molecular and Functional Imaging, Jinan University, Guangzhou, Guangdong China; 4 Yuanxi Biotech Inc., Guangzhou, Guangdong China; 5 Guangdong Landau Biotechnology Co. Ltd., Guangzhou, Guangdong China; 6 Institute of Automation, Chinese Academy of Sciences, Beijing, China; 7 Department of Genetics, Yale University School of Medicine, New Haven, CT USA

**Keywords:** Developmental biology, Mechanisms of disease

## Abstract

Autism spectrum disorder (ASD) is a complex neurodevelopmental condition that affects social interaction and behavior. Mutations in the gene encoding chromodomain helicase DNA-binding protein 8 (CHD8) lead to autism symptoms and macrocephaly by a haploinsufficiency mechanism. However, studies of small animal models showed inconsistent findings about the mechanisms for CHD8 deficiency-mediated autism symptoms and macrocephaly. Using the nonhuman primate as a model system, we found that CRISPR/Cas9-mediated *CHD8* mutations in the embryos of cynomolgus monkeys led to increased gliogenesis to cause macrocephaly in cynomolgus monkeys. Disrupting *CHD8* in the fetal monkey brain prior to gliogenesis increased the number of glial cells in newborn monkeys. Moreover, knocking down *CHD8* via CRISPR/Cas9 in organotypic monkey brain slices from newborn monkeys also enhanced the proliferation of glial cells. Our findings suggest that gliogenesis is critical for brain size in primates and that abnormal gliogenesis may contribute to ASD.

## Introduction

Autism spectrum disorder (ASD) comprises a group of neurodevelopmental conditions that affect social interaction and communication and display repetitive behavior. The gene encoding chromodomain helicase DNA-binding protein 8 (CHD8), an ATP-dependent chromatin remodeling factor that regulates gene transcription, has been described as an autism susceptibility/intellectual disability gene, as mutations in *CHD8* were found to be strongly associated with ASD^1–6^. Individuals with heterozygous CHD8 mutations display various clinical features consisting of autistic behaviors, macrocephaly, and facial dysmorphisms^6–10^.

Because heterozygous *CHD8* mutations were identified in ASD patients, the haploinsufficiency of the *CHD8* gene is thought to account for the pathogenesis of CHD8-related ASD. In support of this theory, mice carrying heterozygous *Chd8* mutations show autistic-like phenotypes and macrocephaly^11–13^ whereas homozygous *Chd8* mutations cause embryonic lethality^14^. However, the behavioral phenotypes in mouse models are heterogenous and vary to a great extent. For example, social deficits were found in some *Chd8* mutant mice^11–13^ but not others^15,16^ whereas repetitive behavior could occur^16^ or was absent^12,13,15^ in Chd8 deficient mice. In addition, the mechanism underlying CHD8 deficiency-mediated macrocephaly remains controversial. Although increased neurogenesis was reported to be the potential mechanism underlying increased brain volume^15,17,18^, neurogenesis was also found to be decreased^11^ or not altered^13^ in mice or mouse neural progenitor cells with Chd8 deficiency. Furthermore, increases in brain volume were observed in female but not male mice carrying a *CHD8* mutation^16^. A potential role for CHD8 in gliogenesis has so far remained unexplored. Because of the lack of pathological studies of the brains of *CHD8* mutant patients, there is no histological evidence for a correlation of abnormal neuronal development with macrocephaly and clinical phenotypes in patients carrying *CHD8* mutations.

Our recent studies of nonhuman primate models of brain diseases indicate that there are substantial differences in brain pathological changes between rodent and primate models^19–23^. For example, loss of the *SHANK3* or *PINK1* gene can cause striking neuronal loss in the monkey brain, a phenomenon that has not been found in the *Shank3* or *Pink1* knockout mice^19,23^. Rodents exhibit many key features of the development and formation of the mammalian brains, which are characterized by the initial neurogenesis followed by the sequential production of glia. However, the timing and sources of proliferative cells for cerebral expansion are considerably different between rodents and primates^24–27^. A noticeable difference is that rodent brains lack gyrification or the folding of the cortical surface, a unique structure seen in large mammals and primate brains^28^. Gliogenesis was found to correlate with enlargement and gyrification of the primate cerebrum^29^, but this interesting finding remains to be verified with genetic evidence. Thus, it is important to establish a nonhuman primate model with *CHD8* mutations to investigate how a *CHD8* mutation affects the development of the primate brain.

In this study, we used CRISPR/Cas9 to target the *CHD8* gene in the embryos of cynomolgus monkeys (*Macaca fascicularis*). As expected, *CHD8* mutations affected early development and led to a low yield of live monkeys. Despite this, analysis of the brain tissues of aborted and stillborn monkeys and the behaviors of the live monkey revealed that *CHD8* mutation abnormally increased gliogenesis to enlarge brain size and to alter social interaction and communication. To avoid embryonic lethality, we disrupted the *CHD8* gene in fetal monkey brains prior to gliogenesis and in brain slices from newborn monkeys, resulting in an increased number of glial cells. These findings together show that in the primates, *CHD8* mutations may primarily increase gliogenesis to cause macrocephaly and autism symptoms, providing a new insight into ASD pathogenesis.

## Results

### Generation and validation of *CHD8* mutant monkeys

**Fig. 1:**
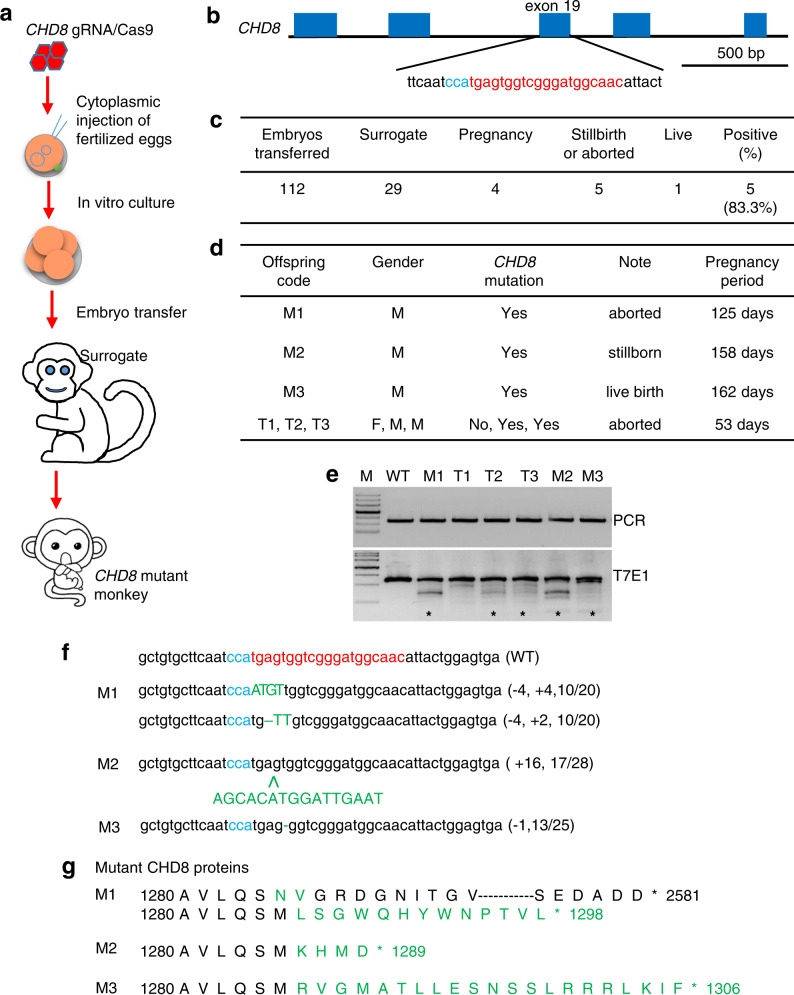
CRISPR/Cas9-mediated *CHD8* mutations in cynomolgus monkeys. **a** One-cell stage embryos of cynomolgus monkeys were injected with *CHD8* gRNA and Cas9 mRNA. Embryos at 4–8-cell stage were transferred to surrogates to produce newborn monkeys with *CHD8* mutations. **b** Schematic diagram of gRNA targeting exon 19 of *CHD8*. Part of the *CHD8* gene structure was shown, Protospacer-adjacent motif (PAM) sequence is highlighted in blue. gRNA targeting site is highlighted in red. **c** The numbers of transferred embryos, surrogates, and pregnancies for generating monkeys with *CHD8* mutations. **d** Summary of generated monkeys carrying *CHD8* mutations. **e** Examination of *CHD8* mutations in monkey offspring by PCR and T7E1 cleavage assay. M DNA marker; WT wild type. Brain tissues of M1, M2, and WT control, embryos from T1, T2, and T3 and umbilical cord from M3 were analyzed. The upper panel shows PCR products and the low panel shows DNA products after T7E1 digestion. Stars indicate DNA mutations. **f** Sequences of mutated *CHD8* alleles in monkey offspring. PAM sequence is highlighted in blue; gRNA targeting site in red. Mutation sequences are marked in green. Insertions (+) or deletions (–) are indicated within parentheses. The numbers of DNA clones showing mutations of the total number of examined clones are also included in parentheses. **g** Identified *CHD8* mutations result in truncation of the CHD8 protein. Amino acid sequences in yellow are present in wild-type CHD8 whereas sequences in green are mutated.

We used the embryonic targeting strategy to delete the *CHD8* gene in fertilized monkey eggs (Fig. 1a). We designed gRNA to target exon 19 of *CHD8*, as exon 19 is shared by two *CHD8* isoforms (NM_001170629.1 and NM_020920.3) (Fig. 1b and Supplementary Fig. S1a). Thus, a frameshift mutation or truncation in this exon is sufficient to disrupt CHD8 expression. After validating that gRNA was able to reduce CHD8 in cultured HEK 293 cells (Supplementary Fig. S1b, c), we injected *CHD8* gRNA with Cas9 to one-cell stage embryos of cynomolgus monkeys using the same methods described in our previous studies^19,21,23^. It is known that only heterozygous mutations of *CHD8* were seen in humans and that homozygous *Chd8* mutations caused embryonic lethality in mice^14^. In our initial studies, we used different *CHD8* gRNAs that might have higher targeting efficiency and failed to obtain any pregnancy (0/41 surrogates). We then used the gRNA described in Fig. 1a and obtained four (4/29) pregnant monkeys after transplanting 112 embryos into 29 surrogate mothers (Fig. 1c). It is likely that the potential lethality of homozygous or extensive mutations yielded a low rate of pregnancies. The four pregnancies we had obtained included a triplet pregnancy that was terminated to yield three aborted fetuses (T1, T2, and T3) at 53 days after embryo transfer. The other three pregnant monkeys produced one aborted fetus (M1) on day 125, one stillborn monkey (M2) that had developed full-term (158 days), and one live offspring (M3) that had been living for 54 months (Fig. 1d). The low birth rate of *CHD8* mutant monkeys supports the idea that CHD8 is essential for early development.

All the offspring were genotyped for mutations in exon 19 of *CHD8* (Fig. 1e, f). The gender of triplets (T1, T2, T3) was determined by the presence of the sex-determining region Y (*SRY*) gene on the Y chromosome^30^. We used deep sequencing to analyze aborted triplets (T1, T2, T3), which showed low rates (5.1%–27.5%) of *CHD8* mutations (Supplementary Fig. S2a). Thus, their fetal loss is unlikely due to *CHD8* mutations because triplet fetuses in macaque monkeys are often miscarried. For M1, M2, and M3, their targeted DNAs were isolated by PCR and cloned for sequencing. Various mutations (–4/+4, –4/+2, +16, and –1) in *CHD8* exon 19 of mutant offspring were identified by DNA sequencing (Fig. 1f). M1, which was miscarried at 125 days of pregnancy, carried bi-allelic mutations, in which one allele had 4 bp deletion and insertion (–4/+4) and the other had –4/+2 bp mutation in its brain (Fig. 1f). Such mutations resulted in two amino acid alternations (M1285N and S1286V) and a frameshift mutation to generate a truncated protein with 1298 amino acids (p.S1286fsX1299) (Fig. 1g). M2 had heterozygous +16 bp mutant sequences in the brain, leading to the truncation at 1289 amino acid position (Fig. 1f, g). The live M3 (male) has heterozygous –1 bp mutation in its umbilical cord and blood sample, which can lead to truncation of CHD8 protein with 1308 amino acids (p.S1286fsX1307), though the nature of *CHD8* mutation in the brain remains to be determined (Fig. 1f, g).

To understand the mosaic nature of *CHD8* mutant monkeys, we used DNA cloning and sequencing to analyze mutation rates in PCR products from various tissues. M1 monkey tissues showed more extensive mutations (>72.7%) than M2 monkey tissues (31.3%–73.7%) (Supplementary Fig. S2b). The more severe mutations in M1 may explain why M1 was terminated earlier than M2. To analyze the potential off-targets, we performed whole-genome sequencing of the blood sample of the live monkey (M3), and the results did not reveal obvious off-target events (Supplementary Fig. S3a). Whole-genome sequencing of the brain cortex DNAs from M1 and M2 did not detect any off-target events in the potential targeting sequences containing no more than five mismatched nucleotides, while M3 had one potential off-target (Supplementary Fig. S3b).

### Increased gliogenesis in *CHD8* mutant monkeys

**Fig. 2:**
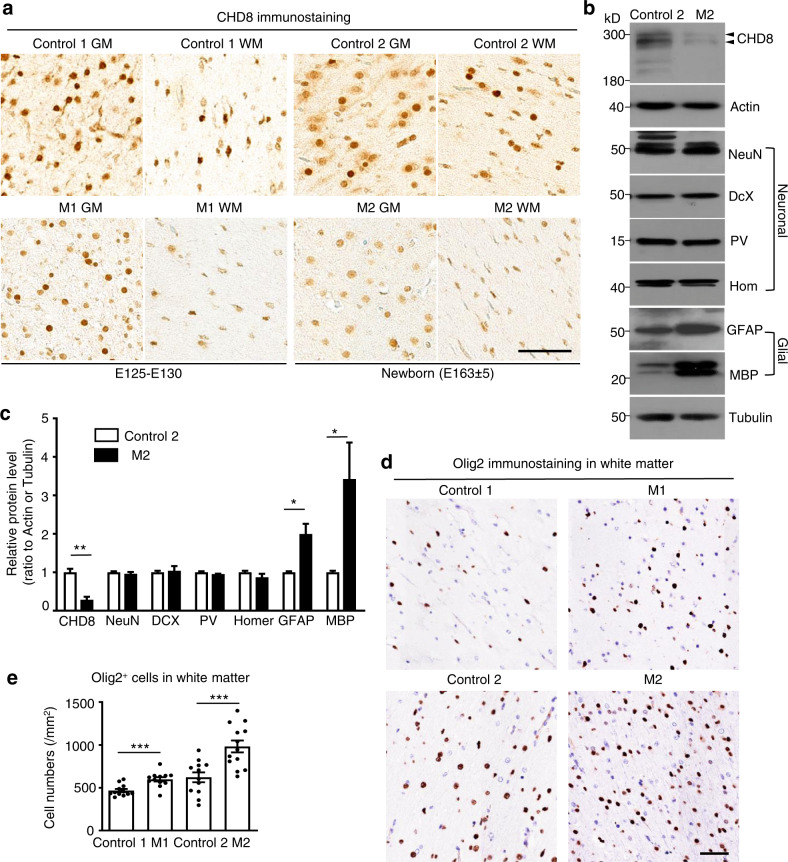
*CHD8* mutant monkeys have decreased CHD8 expression, increased glial marker levels and normal neuronal marker levels. **a** Immunostaining of the prefrontal cortex gray matter (GM) and white matter (WM) of control and *CHD8* mutant monkeys (M1, M2) with anti-CHD8 antibody. CHD8 shows a higher expression in E125–E130 than newborn (E163 ± 5). Scale bar: 50 μm. **b** Western blotting of the prefrontal cortex of the newborn monkeys (control-2 and M2). The blots were probed with antibodies to neuronal proteins (NeuN, DcX, PV, and Hom) and glial proteins (GFAP, MBP). Tubulin served as a loading control. **c** Quantification of the ratios of neuronal and glial proteins to the loading control on western blots. The data were obtained from four western blotting experiments and are presented as means ± SEM. *P < 0.05; **P < 0.01. **d** Immunostaining of the white matter of control and *CHD8* mutant monkeys with an antibody to olig2, a marker protein for oligodendrocytes. The nucleus was stained with hematoxylin. Scale bar: 50 μm. **e** Density of oligodendrocytes in the white matter of control and *CHD8* mutant monkeys. The data were obtained by counting 12 images and presented as means ± SEM per mm^2^. ***P < 0.001.

The aborted M1 monkey provided us with tissues for immunocytochemical studies, and the stillborn M2 monkey offered more tissues for both immunocytochemical and western blotting analysis. We also obtained wild-type monkey (control-1) at E130 and a newborn wild-type (control-2) monkey for comparing with M1 and M2, respectively. Immunohistochemistry validated that *CHD8* mutations could reduce the immunostaining of CHD8 at the protein level in the brains of M1 and M2 (Fig. 2a). We then used western blotting to analyze the expression of neuronal and glial proteins in control-2 and M2 monkey brain tissues. The results revealed striking increases in glial fibrillary acidic protein (GFAP) and myelin basic protein (MBP), which are marker proteins for astrocytes and oligodendrocytes, respectively, in the M2 monkey brain. However, examination of several neuronal proteins (NeuN, parvalbumin (PV), Homer (Hom), and doublecortin (Dcx)), did not show any significant difference between control-2 and M2 monkeys (Fig. 2b). Quantification of the ratios of examined proteins to the loading control tubulin on multiple western blots verified that *CHD8* mutations selectively increased glial proteins but not neuronal proteins (Fig. 2c). Glia are found in both gray and white matter, but there are more oligodendrocytes in white matter^31^. We, therefore, used immunocytochemical staining of the white matter to identify oligodendrocytes with an antibody to olig2, a protein that is specifically expressed in oligodendrocytes. This examination also showed that there were more oligodendrocytes in the white matter of M1 and M2 (Fig. 2d, e).

**Fig. 3:**
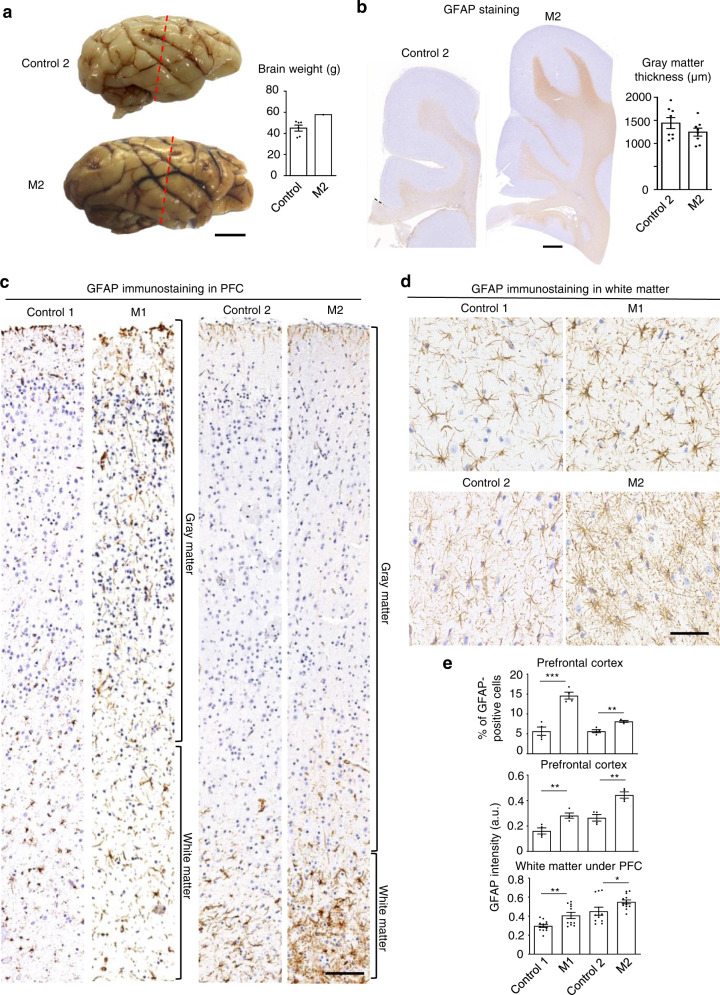
Macrocephaly and increased glial cells in newborn *CHD8* mutant monkeys. **a** Photographs of control-2 and M2 monkey brains. The brains were sliced at the same position (red lines) for immunostaining. Quantification of brain weights of M2 and six age-matched controls is shown on the right panel. Scale bar: 1 cm. **b** GFAP immunostaining of the white matter in control and *CHD8* mutant monkeys. The thickness of gray matter, which is GFAP-negative, in the cortical regions of M2 and age-matched control-2 is shown on the right panel (*n* = 8 measures in each animal). Scale bar: 1 mm. **c** GFAP immunostaining of the entire cortical layers of control and *CHD8* mutant monkeys. GM gray matter; WM white matter. Scale bar: 50 μm. **d** Large magnification micrographs of GFAP immunostaining in the white matter of control and *CHD8* mutant monkeys. Scale bar: 20 μm. **e** Quantification of the density of GFAP-positive cells from GFAP-immunostaining results. The data were obtained by counting 4–11 images in each group and presented as means ± SEM per mm^2^. *P < 0.05; **P < 0.01, ***P < 0.001.

The brains of newborn monkeys have been well developed and allowed us to compare the brain sizes of newborn control-2 and M2. This comparison revealed that the M2 brain was 28% larger in weight with enlarged white matter labeled by immunostaining of GFAP (Fig. 3a, b), which is selectively expressed in astrocytes, another major type of glial cells that are mainly distributed in the white matter. The gray matter thickness appears normal (Fig. 3b, c). Immunostaining of the entire monkey cortex layers clearly demonstrated more astrocytic staining in the cortex of *CHD8* mutant monkeys (M1 and M2) than the control monkey cortex (Fig. 3c). Quantification of the numbers of astrocytes and GFAP-immunostaining intensity in the white matter verified that *CHD8* mutations increased GFAP labeling (Fig. 3d, e). However, immunostaining of Iba1, a marker protein for microglial cells, did not show different staining between the control and *CHD8* mutant monkeys (Supplementary Fig. S4a, b). In addition, the control and *CHD8* mutant monkey brains showed no difference in immunostaining of neurons with an antibody to the neuronal marker NeuN (Supplementary Fig. S5a–c). In other brain regions such as the cerebellum, neuronal staining by antibodies to neuronal proteins (NeuN and calbindin) did not show any obvious difference between control-2 and M2 (Supplementary Fig. S5d). Thus, *CHD8* mutations appear to selectively increase astrocytic and oligodendrocytic protein expression.

**Fig. 4:**
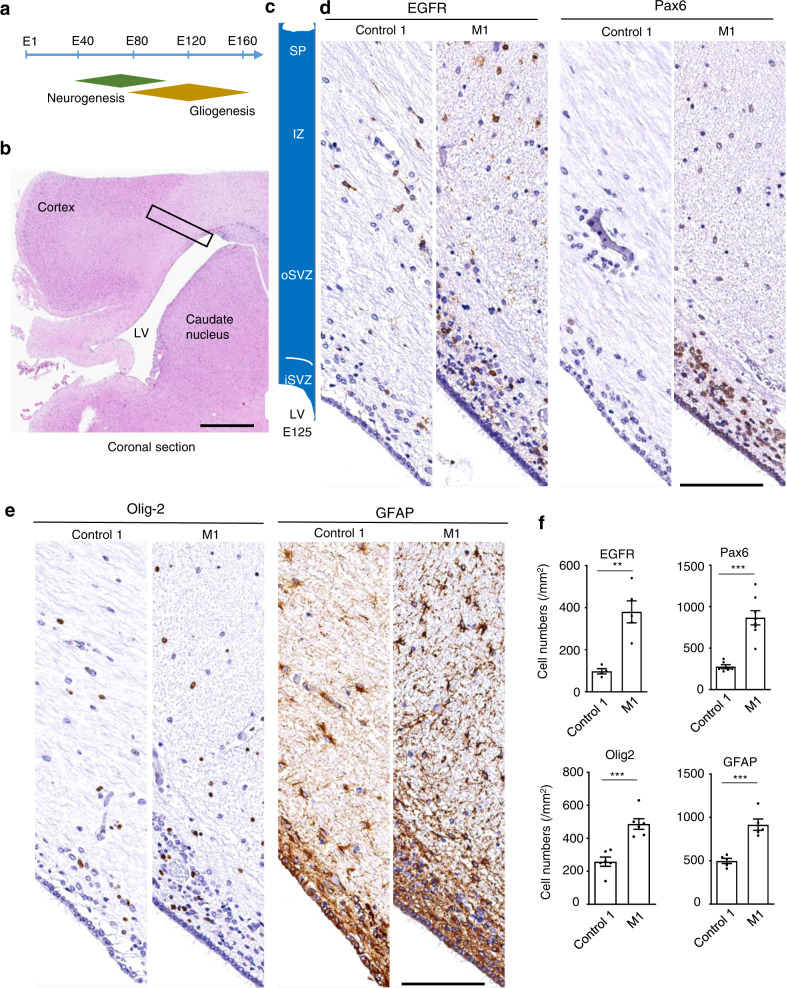
Increased gliogenesis in the developing brain of *CHD8* mutant monkey M1. **a** The timeline for embryonic neurogenesis and gliogenesis in the macaque monkey. **b** The coronal section of the control-1 monkey (E130) showing the cortex, caudate, corpus callosum and LV. Rectangle indicates the location of brain slices that were used to examine the staining and numbers of neuronal and glial cells. Scale bar: 1 mm. **c** The brain slice used for examination consists of different lays including the inner subventricular zone (iSVZ), outer subventricular zone (oSVZ), intermediate zone (IZ), and subplate (SP). **d** Immunostaining of progenitor cells with antibodies to EGFR and Pax6. The nucleus was stained with hematoxylin. Scale bar: 100 μm. **e** Immunostaining of the white matter near the LV with antibodies to olig2 and GFAP, respectively. Scale bar: 100 μm. **f** Quantification of the relative numbers of EGFR- and Pax6-positive cells in iSVZ and the numbers of oligodendrocytes and astrocytes in the brain slices. The data were obtained by counting 4–7 brain slices per group and presented as means ± SEM. **P < 0.01; ***P < 0.001.

Since astrocytes and oligodendrocytes are derived from the same progenitor cells (radial glial cells) that also give rise to neurons before gliogenesis, the lack of NeuN alteration in M1 and M2 suggests that *CHD8* mutations selectively increased the proliferation of astrocytes and oligodendrocytes. M1 monkey allowed us to explore this issue, as it was terminated at E125, a time after neurogenesis (E45–E102) and during gliogenesis (E90–E145) in the macaque monkey brain^29,32^ (Fig. 4a). Subventricular zone (SVZ) is critical for neurogenesis and gliogenesis in the mammalian brains and is divided into the inner (iSVZ) and outer (oSVZ) layer in the primate brains. oSVZ, which is absent in the rodent brain, is responsible for the expansion of the cerebral cortex in the primates^25,33^. Thus, we used brain slices containing iSVZ and oSVZ in the control-1 (E130) and M1 (E125) monkeys to assess the distribution of marker proteins for neurogenesis and gliogenesis (Fig. 4b, c). Examination of the progenitor markers such as Pax6 and EGFR revealed that Pax6-positive cells were mainly localized in iSVZ whereas EGFR-positive cells were distributed in all layers, which are consistent with results previously reported^29^. Importantly, more Pax6- and EGFR-positive cells were present in M1 than control-1 (Fig. 4c). These results suggest that more progenitor cells are in M1 for developing to neuronal or glial cells. However, anti-NeuN immunostaining showed weak or absent staining in the region of iSVZ and oSVZ in both control-1 and M1 (Supplementary Fig. S6a), suggesting that neurogenesis had been largely completed. In contrast, staining with antibodies to glial cells (olig2 and GFAP) revealed a number of positive cells in iSVZ and oSVZ. M1 displayed notably more olig2 and GFAP-positive cells than control-1 (Fig. 4d). This important difference was verified by quantitation of the density of progenitor cells and glial cells in the brain slices (Fig. 4e). We also examined the cerebellum but did not find any obvious difference for NeuN and GFAP staining between control-1 and M1 (Supplementary Fig. S6b). Postnatal gliogenesis occurs in the mammalian brains^34,35^. Examining the cortex near the lateral ventricle (LV) region revealed more glial cells and enlarged white matter in M2 than control-2 (Supplementary Fig. S7). Taken together, gliogenesis in the cerebral cortex is obviously enhanced in *CHD8* mutant monkeys when compared with the control monkeys.

**Fig. 5:**
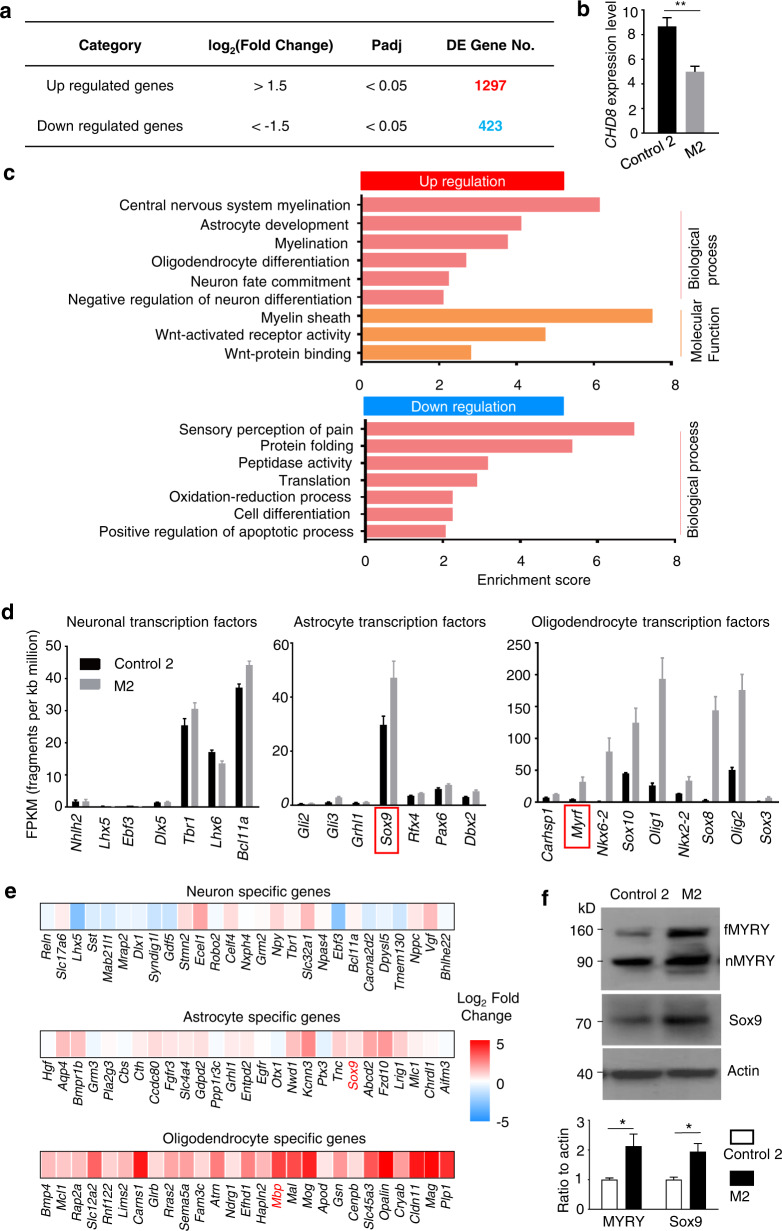
RNA-seq analysis of the prefrontal cortex (PFC) of control-2 and M2 monkeys reveals upregulation of genes for myelination and glial function. **a** Summary of differentially expressed (DE) genes in M2 PFC compared to control-2 PFC. Significance cutoffs correspond to log2 (fold change) > 1.5 and adjusted *P* value (*P*adj) < 0.05. **b** Reduced *CHD8* expression in M2 brain cortex in RNA-seq analysis. **P < 0.01. **c** Representative significant gene ontology (GO) terms for DE genes of up- and downregulation. Odds ratio > 2 and *P*adj < 0.05. **d** Expression levels of enriched transcription factors for neurons, astrocytes and oligodendrocytes demonstrate upregulation in gliogenesis, but not neurogenesis. The data were obtained by averaging three samples and presented as means ± SEM. **e** Heatmap analysis of neuron, astrocyte, and oligodendrocyte-specific genes revealing upregulation in oligodendrocyte-enriched genes but not in neuron-enriched genes. Fold change was plotted on a log_2_ color scale, with blue representing lower expression and red representing higher expression in M2 than control-2. **f** Western blotting showing the increased expression of full-length (fMYRF), N-terminal (nMYRF) MYRF and Sox9 in the brain cortex of M2 when compared with control-2. The ratios (means ± SEM) of MYRF and Sox9 to the loading control actin were obtained by quantifying the intensity of MYRF, Sox9, and actin on three independent western blots.

M2 monkey brain tissues also allowed us to perform RNA-seq to compare gene expression profiling with control-2, which could provide complementary information regarding the expression of neuronal and glial genes. We examined the expression of 18,224 genes in our data analysis by comparing RNA-seq results from the prefrontal cortex of control-2 and M2 and found 1298 up- and 423 downregulated genes above the threshold (Fig. 5a, b). In Gene Ontology analysis, upregulated genes were enriched in myelin-related processes, astrocyte and oligodendrocyte development/differentiation, and Wnt-signal pathway (Fig. 5c). Because of differential expression of neuronal and glial proteins in *CHD8* mutant monkey brains, we focused on marker genes and transcription factors of neuronal and glial cells based on the published information for these genes^36^. Transcription factors specific for oligodendrocytes were upregulated to a greater extent than those for astrocytes whereas neuronal transcription factors were expressed at similar levels in control-2 and M2, consistent with greater increases in oligodendrocytic gene expression (Fig. 5d, e). Major oligodendrocyte transcription factors, including *Olig2, Olig1, MYRF, Sox10, Sox8, Sox3, Nkx2-2, Nkx6-2*, and *Carhsp1*, were significantly upregulated with fold change ranging from 1.7 to 74 in M2, indicating enhanced oligodendrogenesis (Fig. 5d, e). Since we previously investigated *Myrf*, an oligodendrocyte-specific transcription factor, in Huntington disease mice^37^, we examined MYRF expression via western blotting using a commercially available antibody. The results confirmed that both full-length and N-terminal MYRF were increased in the brain cortex of M2 monkey (Fig. 5f). Thus, RNA-seq results support the findings that the brain cortex in M2 has increased expression of glial proteins, particularly oligodendrocytic proteins, which were also revealed by western blotting and immunohistochemistry (Figs. 2 and 3).

However, RNA-seq analysis of bulk tissues would be difficult to define gene alterations in specific types of cells or in specific regions. The proliferation of glial cells in SVZ in the primate brain is essential for the expansion and gyrification of the primate cerebrum^29^. We used immunostaining to examine the distribution of cells expressing Ki67, a proliferation marker, in the CHD8 mutant monkey brains. We found there was a significant increase in Ki67-positive cells in SVZ, but not in white matter, in M1 and M2 monkeys as compared with controls (Supplementary Fig. S8). This result suggests that increased gliogenesis in *CHD8* mutant monkeys is more likely due to the enhanced proliferation of glial progenitor cells in SVZ rather than the proliferation of differentiated glial cells in the white matter.

### More glial cells by knocking down *CHD8* in the brains of fetal and newborn monkeys

**Fig. 6:**
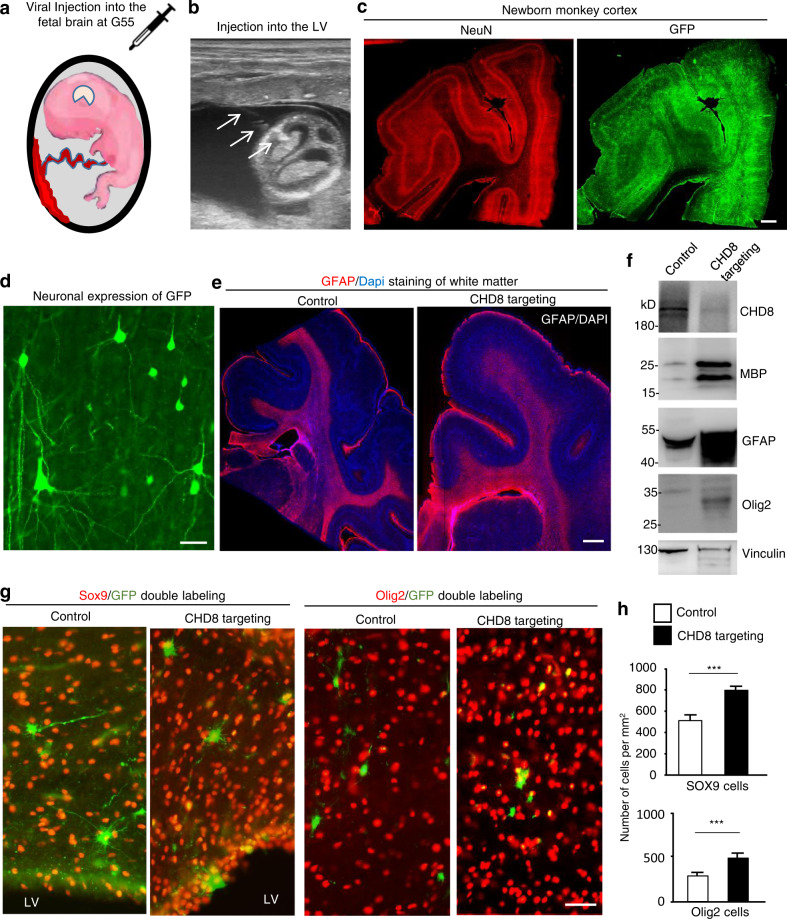
More glial cells in the newborn monkey brain after LV injection of lentivirus targeting CHD8 at G55. **a** In utero delivery of lentiviral CHD8 gRNA/Cas9 into the LV of fetal monkey brain at ~G55. **b** Ultrasound image of injection of lentivirus into the LV of the fetal monkey brain at G55. Arrows indicate a needle. **c** Widespread expression of GFP, which reflects lentiviral *CHD8* gRNA/Cas9 transduction in the brain of newborn monkey. Scale bar: 1 mm. **d** Neuronal expression of GFP in the newborn monkey brain cortex. Scale bar: 10 μm. **e** GFAP labeling of the white matter of the brain cortical region in control and CHD8-targeted newborn monkeys. Scale bar: 1 mm. **f** Western blotting of the lentiviral-infected brain regions showing that *CHD8* gRNA/Cas9 expression reduced CHD8 expression and increased levels of glial proteins (Sox9 and Olig2). **g** Double immunostaining of lentiviral-infected brain regions showing that *CHD8* gRNA/Cas9 expression increased Sox9-labeled astrocytes and Olig2-labeled oligodendrocytes when compared with control gRNA/Cas9 expression. Scale bar: 20 μm. **h** Quantitation of the relative number of oligodendrocytes and astrocytes. The data were obtained from one newborn monkey targeted by control gRNA/Cas9 and two newborn monkeys targeted by *CHD8* gRNA/Cas9. Brain sections (1.3 mm apart from each other) from the brain cortical region in the control gRNA/Cas9 (six sections) and CHD8 gRNA/Cas9 (eight sections) targeted monkeys were used for quantitative analysis. At least four images (×20) from each brain section were taken to obtain the average numbers of glial cells per mm^2^. The data are presented as means ± SEM (*n* = 24 images for control gRNA/Cas9 and *n* = 32 images for *CHD8* gRNA/Cas9). ***P < 0.001.

The facts that homozygous *CHD8* deletion is embryonic lethal in mice and that only heterozygous *CHD8* mutations are seen in patients explain the low rate of survived *CHD8* mutant monkeys in the CHD8 gene-targeting at the one-cell embryo stage. Transcription profiling studies indicate that in the monkey brain, neurogenesis peaks around gestation day 55 (G55) and continues until G80, which overlaps the beginning of gliogenesis^38,39^. To more rigorously investigate whether *CHD8* expression is important for gliogenesis, we generated lentiviral vectors to express *CHD8* gRNA and Cas9 (Supplementary Fig. S9a) and performed in utero delivery of lentiviral *CHD8* gRNA/Cas9 into the fetal monkey brain at ~G55 (Fig. 6a). For the control, we used lentiviral control RNA/Cas9 that did not target any specific gene. The pregnancy was assessed by ultrasound examination, and injection of lentiviruses (50 μL lentiviral control or *CHD8* gRNA mixed with 100 μL lentiviral Cas9, viral titer 1 × 10^9^ vg/mL) into the LV of the fetal monkey brain was performed under the guidance of continuous ultrasound imaging (Fig. 6b and Supplementary Fig. S9b). We injected lentiviral control gRNA/Cas9 into one fetus, and lentiviral CHD8 gRNA/Cas9 into two fetuses, all at ~G55. All these three fetuses developed to newborn monkeys that were naturally delivered around days 161–164. The brains of these newborn monkeys were isolated at postnatal day 7–8 and revealed that lentiviral transduction was widespread in the cortical layers, as lentiviral vectors also expressed GFP (Fig. 6c). Lentiviral transduction led to the expression of transgene, reflected by GFP expression, in neuronal cells with normal morphology (Fig. 6d). The white matter, which was clearly labeled by anti-GFAP, appeared to be larger in *CHD8-*targeted monkey brain than in the control monkey brain (Fig. 6e and Supplementary Fig. S9c). Importantly, western blotting analysis of lentiviral-infected brain regions showed that CHD8 was decreased, accompanied by elevated levels of Olig2 and SOX9, which are marker proteins for oligodendrocytes and astrocytes (Fig. 6f).

It is important to examine whether the density of glial cells was increased in the white matter of *CHD8*-targted monkey brain. Thus, we performed double immunofluorescent staining to verify that lentiviral *CHD8* gRNA/Cas9 was expressed in both neuronal and glial cells (Supplementary Fig. S10a) and was able to reduce the level of CHD8 (Supplementary Fig. S10b). While CHD8 targeting did not influence neuronal processes, morphology, and density (Fig. 6d and Supplementary Fig. S11a), astrocytic proliferation, which was revealed by anti-GFAP labeling, appeared to be increased (Supplementary Fig. S11b). However, GFAP labeling could not clearly distinguish individual cells. We therefore used anti-SOX9 to label the nuclei of astrocytes and anti-olig2 to identify oligodendrocytes. Virus-mediated transgene (GFP) expression can decline overtime during brain development but disrupting the *CHD8* gene by CRISPR/Cas9 in the brain cells including neuronal and glial progenitor cells is permanent. Thus, to assess the effect of targeting *CHD8* gene at G55 on the glial cells in the newborn monkeys, we identified the brain regions expressing GFP in the newborn monkeys and counted glial cell numbers in the viral infected brain regions. The results demonstrated an increase in the density of astrocytes and oligodendrocytes in *CHD8*-targeted brain region as compared with control lentiviral infection (Fig. 6g and Supplementary Fig. S12). Quantitation of the relative numbers of glial cells in the examined areas also confirmed that targeting *CHD8* by CRISPR/Cas9 at G55 could increase the numbers of astrocytes and oligodendrocytes in the newborn monkey brains (Fig. 6h).

**Fig. 7:**
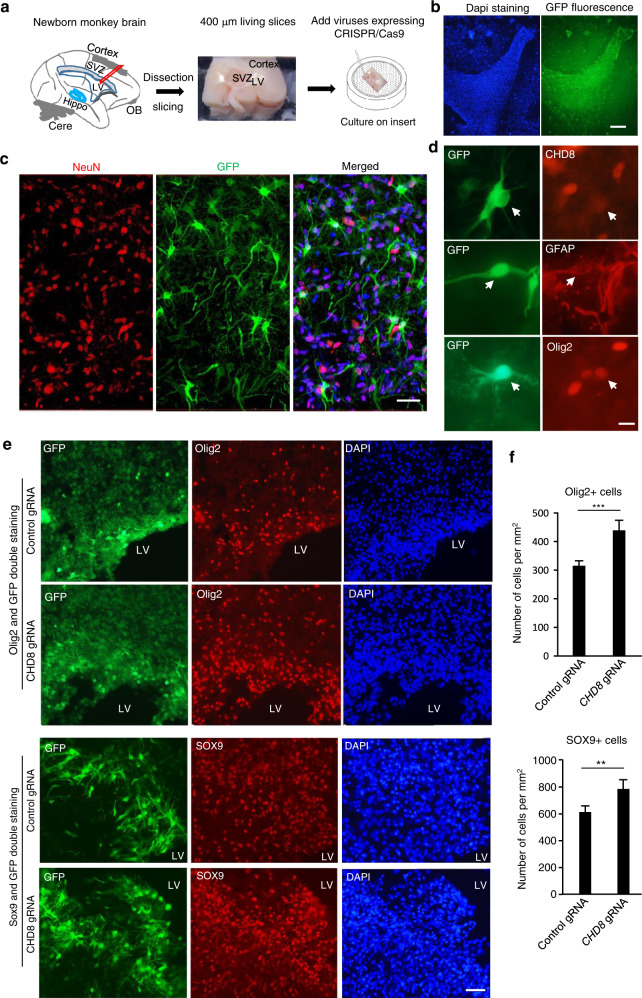
More glial cells after CHD8 targeting in brain slice culture system. **a** Cortical brain region near the LV and SVZ in the newborn monkeys was isolated for brain slice culture. OB olfactory bulb, Hippo hippocampus, Cere cerebellum. **b** After infection with lentivirus for 10 days, the brain slices showed strong GFP signaling, reflecting lentiviral transduction. Scale bar: 100 μm. **c** Immunostaining of the infected brain slices showing that GFP-positive cells contain NeuN positive and negative cells. Scale bar: 25 μm. **d** Double immunostaining showing that lentiviral *CHD8* gRNA/Cas9 expression, reflected by GFP expression, reduced CHD8 expression and occurred in astrocytes and oligodendrocytes. Scale bar: 10 μm. **e** Double immunostaining of the brain slice region near the LV showing that the density of olig2-labeled oligodendrocytes and SOX9-labeled astrocytes was increased in the lentiviral *CHD8* gRNA/Cas9 infected region as compared with lentiviral control gRNA/Cas9 infection. Scale bar: 25 μm. **f** Quantitation of the relative numbers of oligodendrocytes and astrocytes. Nine brain slices from three newborn monkeys were used for statistical analysis. The data are presented as means ± SEM. **P < 0.01; ***P < 0.001.

Transcriptional profiling studies also showed that gliogenesis peaks after birth and continues through childhood in the primates^38,39^. To obtain additional evidence to support the idea that CHD8 plays an important role in gliogenesis in the primate brain, we used brain slices from newborn monkeys to examine the effect of *CHD8* deficiency on glial cells after birth (Fig. 7a). The brain region containing the LV was infected by lentiviral *CHD8* sgRNA/Cas9 (Supplementary Fig. S13a), as these regions contain glial cells that can proliferate and migrate to the cortical layers, a process important for enlarging brain size in the primates^29^. Under ex vivo culturing conditions, glial cells were preferentially infected by lentiviral vectors, and most of GFP-positive cells were negative to NeuN labeling despite use of the EF1-α promoter to express GFP (Fig. 7b, c), allowing us to examine the effect of targeting *CHD8* on the proliferation of glial cells. Double immunofluorescent staining revealed that lentiviral infection apparently led to the expression of *CHD8* gRNA in oligodendrocytes and astrocytes (Supplementary Fig. S13b) and decreased CHD8 expression in GFP-positive cells (Fig. 7d). Immunostaining of the brain region near the LV demonstrated the increased numbers of oligodendrocytes and astrocytes, which could be readily identified by the nuclear labeling of olig2 and SOX9, respectively (Fig. 7e).

### Enlarged white matter and macrocephaly in the live *CHD8* mutant monkey

**Fig. 8:**
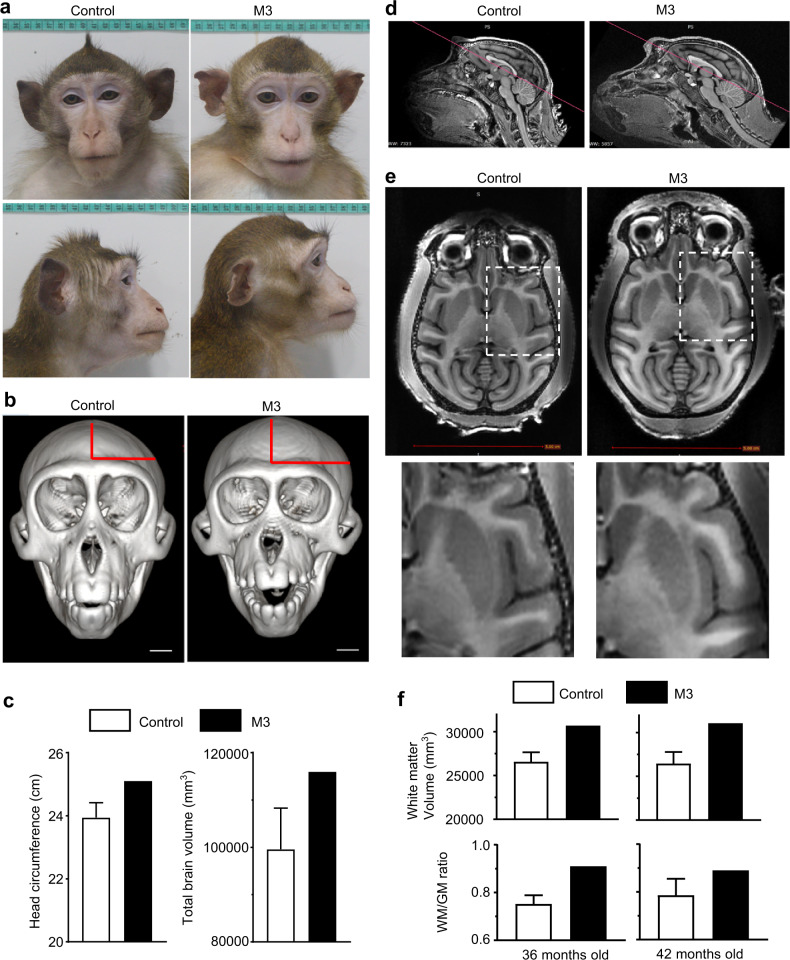
Macrocephaly and enlarged white matter in adult *CHD8* mutant monkey M3. **a** Head and face photos of M3 and an age-matched control monkey at 54 months. **b** Skeleton head images of M3 and control monkey. The sizes (height and width) of the head are indicated by red lines. Scale bar: 1 cm. **c** Head circumference (cm) and total brain volume (mm^3^) of M3 and 15 age-matched male control monkeys at 36 months. **d** Sagittal MRI images of M3 and a control monkey. The red lines indicate the same location and orientation of the slices that were used to obtain coronal MRI images containing the cerebral cortex. **e** Horizontal MRI images of M3 and control monkey showing white matter that appears whiter and gray matter that is darker. Scale bar: 5 cm. The coronal images were obtained from the respective brain slices shown in (**d**). The enlarged images in the dashed square are shown beneath each coronal image. M3 brain shows enlarged white matter in several lobes shown. **f** Quantitation of the volume of white matter and the ratio of white matter to gray matter. Controls consist of three age-matched monkeys. The data are presented as means ± SEM.

To examine whether *CHD8* mutations caused macrocephaly, we carefully examined the brain size of the CHD8 mutant monkeys (M2 and M3). We noticed a greater weight of the isolated M2 brain (57.8 g) than controls (45 ± 2.8 g, *n* = 6), supporting 28% larger brain size in M2 at birth (Fig. 3a). This finding, along with increased gliogenesis in M1 and more glial cells in M2, led us to carefully examine whether the live M3 monkey displayed any abnormal size of its head. By comparing with 15 age- and body weight-matched male monkeys at 24 months of age, we found the size of M3’s head is apparently larger than the age- and sex-matched control monkeys. Figure 8a shows the photos of M3’s head and the control head of an age- and weight-matched male monkey at 54 months of age. Skeletal imaging of heads and measurements of the head circumference (*n* = 15 for control) verified that M3 monkey had a larger head (25.1 cm) than the control monkeys (23.26 ± 0.28 cm, *n* = 15) (Fig. 8b, c). MRI was then performed to examine the brain structure, especially the gray matter and white matter. Total brain volume of M3 monkey (115,916 mm^3^) was larger than that (101,622 ± 3626 mm^3^) of the age-matched control monkeys (*n* = 7) (Fig. 8c). The white matter volume of M3 monkey (36-m-old: 30,690 mm^3^; 42-m-old: 31,052 mm^3^) had a larger volume than that of the control monkeys (36-m-old: 26,413 ± 557 mm^3^; 42-m-old: 36,517 ± 501 mm^3^, *n* = 7). To rule out the influence of individual brain sizes on the relative areas of the white matter, we measured the ratios of white matter to gray matter (WM/GM) on the same brain regions in both controls and M3, which were determined by their imaging positions. This quantification demonstrated that the ratio of WM/GM of M3 monkey (36-m-old: 0.89; 42-m-old: 0.91) was higher than that of the control monkey (36-m-old:0.75 ± 0.02; 42-m-old: 0.78 ± 0.03, *n* = 7) (Fig. 8f). Thus, this imaging analysis revealed that the areas of the white matter were larger in the M3 monkey than the control monkeys (Fig. 8d, e). Considering the increased glial cells in M1 and M2 monkeys, these results suggest that *CHD8* mutations can increase gliogenesis to enlarge white matter, resulting in macrocephaly.

## Discussion

The formation and maturation of the brain are largely determined by neurogenesis and gliogenesis during embryonic and postnatal development. Using the nonhuman primate models to investigate the effect of CHD8 on brain development, we found that CHD8 expression is important for normal gliogenesis because its deficiency can enlarge the brain size via abnormally increased gliogenesis. This finding highlights the specific function of CHD8 and unique pathogenesis of CHD8 deficiency in primates.

In the embryonic brain, both neurons and astrocytes/oligodendrocytes are derived from the common multipotent neuroepithelial cells (NECs) but neurogenesis precedes gliogenesis. However, early brain development in primates has distinct features. Unlike other animals, the primates contain white matter in a greater proportion^40^, and glial cell proportion increases faster than the gray matter in the primate brains^41,42^. Proliferation of progenitor cells occurs in the SVZ, which is small in rodents but much larger and complex in the primates^25,43,44^. Also, the expansion of progenitor cells in the oSVZ leads to cerebral gyrification, a process that is absent in rodents^25,33^. Oligodendrocytes are one of the major types of glial cells that are generated in late embryonic stage. During postnatal development, gliogenesis and myelination continue in the primate brains^38,39,45^.

Our findings provide several lines of evidence to support that *CHD8* mutations enhance gliogenesis in the primate brain. First, western blotting and immunohistological studies of the brains of M1 and M2 monkeys show the elevated expression of glial proteins for astrocytes and oligodendrocytes before birth. Quantification of the numbers of astrocytes and oligodendrocytes in these two mutant monkey brains verified a greater number of glial cells in their brains than the age-matched control monkey brains. However, examining neuronal proteins and numbers did not show any significant differences between *CHD8* mutant and control monkeys, suggesting that neurogenesis is not significantly altered by *CHD8* mutations. Second, in the brain region around LV that was undergoing gliogenesis, we observed much more glial cells and their progenitor cells in M1 than the control monkey. Also, in the brain cortex of newborn monkeys, we observed more glial cells and enlarged white matter in M2 when compared with control monkey. The increased gliogenesis in M1 and M2 and macrocephaly in the live *CHD8* mutant monkey provide critical evidence to support the idea that gliogenesis in the oSVZ, which is absent in rodents, might be responsible for the expansion and folding of the cortical surface in the primates^29^.

In support of the results from embryonic *CHD8-*targeted monkeys, we also conducted experiments to delete the *CHD8* gene in the fetal monkey brains before gliogenesis occurs and in the brain slices from newborn monkeys. The results consistently showed that CHD8 deficiency could increase the numbers of glial cells without influencing neuronal density and differentiation. The more important finding is that targeting CHD8 prior to gliogenesis in monkey fetuses could also increase the number of astrocytes and oligodendrocytes, strongly supporting the idea that CHD8 regulates gliogenesis during early brain development. In addition, transcriptional profiling results from the M2 monkey are in line with the dysfunction of CHD8-mediated gene expression in the CHD8 mutant monkey brain. The effects of *CHD8* mutations on the proliferation and number of glial cells in the macaque monkey are particularly relevant to the macrocephaly phenotype seen in the human brain^6,8–10^. Indeed, the live monkey with *CHD8* mutations showed enlarged white matter as compared with the age-matched control monkeys. Glial proliferation and myelination in the primate brains continue after birth and last until adulthood^38,39^. This unique feature makes nonhuman primates an ideal model to investigate gliogenesis when compared with rodents in which glial proliferation and myelination are largely completed after birth^46,47^. Using brain slices from newborn monkeys, we could examine the proliferation of glial cells and found that CHD8 knockdown also increased the numbers of astrocytes and oligodendrocytes. This in vitro evidence strongly supports the in vivo finding that *CHD8* mutations in monkey embryos led to the increased gliogenesis in the developing monkey brains.

CHD8 is one of the ATP-dependent chromatin remodeling factors that regulate the transcription of a large number of genes^48,49^. Although it remains unknown how CHD8 can differentially regulate gene expression in various types of cells at different developmental stages, it appears that the complex regulation of transcriptional networks by CHD8 may be cell type- and species-dependent. In the rodent progenitor cells, CHD8 can repress the activities of multiple transcription factors for neuronal differentiation such that CHD8 deficiency was thought to increase neurogenesis^15,17^. CHD8 deficiency was further found to affect axonal elongation and neuronal survival^50^. The effects of CHD8 deficiency may be species-dependent, as complete elimination of CHD8 in oligodendrocytes was found to inhibit the differentiation and maturation of oligodendrocytes in the rodent brain^51,52^. However, CHD8 deficiency-mediated suppression of oligodendrogenesis found in mouse models is hardly accountable for enlarged brain size that occurs in the majority of patients with CHD8 mutations. Instead, our studies of the nonhuman primate revealed an increase in gliogenesis caused by CHD8 deficiency. This increased gliogenesis was caused by CHD8 haploinsufficiency rather than complete ablation, because CRISPR/Cas9 targeting leads to knockdown or incomplete depletion effect due to the nature of mosaic mutations, which can mimic heterozygous inactivation of CHD8 in patients, at least to some extent. Since complete elimination of CHD8 can result in embryonic^14^ and postnatal^51,52^ lethality in mice, the extent to which CHD8 is suppressed may have differential effects on neuronal and glial cells as well as their progenitor cells. Indeed, CHD8’s effects as a transcriptional regulator are dosage-dependent^17^. Thus, it is possible that *CHD8* mutation or haploinsufficiency preferentially affects transcription networks or/and signaling pathways for the differentiation of progenitor cells to glial cells and their proliferation in the primate brain.

Due to the complex and sophisticated regulation of gene transcription by CHD8 in different types of cells and at different developmental stages, understanding ASD pathogenesis related to *CHD8* mutations faces considerable challenges. A large number of studies have employed transcription analysis of *CHD8* mutant cellular and animal models but have not reached a conclusion regarding how CHD8 mutations cause macrocephaly^53,54^. Our analysis of gene expression in *CHD8* mutant monkey brains suggests that knocking down CHD8 can upregulate a number of genes that are important for the proliferation of glial cells and increase the proliferation of Ki67-positive progenitor cells in SVZ. The more important evidence would be the increased number of glial cells in the *CHD8* mutant monkey brains, which reflects the outcome of deficiency in the CHD8-mediated complex regulation of gene expression. To validate this important finding, we utilized in utero injection of the fetal monkey brain and brain slices from newborn monkeys to knockdown *CHD8*, and the results support those from the monkey model generated by embryonic *CHD8* targeting.

Another important evidence for increased gliogenesis in the *CHD8* mutant monkey brains is the macrocephaly of the live *CHD8* mutant monkey, which mirrors the enlargement of head size seen in patients with *CHD8* mutations^7–9,55^. The white matter of the cerebral cortex contains all axons that support long-range cortical connectivity and are essential to cognitive function^40^. Germline heterozygous mutations in *PTEN* have been identified in children with both ASD and macrocephaly, and are associated with marked abnormalities in the brain white matter and reduced cognitive ability^56^. Thus, any alterations in the development of either neuronal or glial cells would adversely affect neuronal activities, leading to altered behaviors. Indeed, MRI examination has revealed abnormal activity or morphology of myelin and white matter in the brains of ASD patients^57–60^. Although our findings demonstrated for the first time that CHD8 deficiency enhances gliogenesis in the primate brain and suggest that abnormal gliogenesis may play an important role in *CHD8* mutation-mediated pathogenesis, further mechanistic studies are required to understand how CHD8 deficiency selectively alters gliogenesis. Despite this, the findings suggest that increased gliogenesis could account for the impaired white matter connectivity or function, at least in *CHD8* mutant patients. The findings that *CHD8* mutant monkeys show increased gliogenesis and macrocephaly underscore the importance in investigating the role of abnormal gliogenesis in ASD. These findings also have therapeutic implications for the correction of abnormal gliogenesis to achieve more effective treatments for ASD.

## Materials and methods

### Reagents

**Table:** 

Reagent or resource source identifier				
Antibodies	Source	Cat. #	IHC/WB dilution	Company
CHD8	Rabbit	A301-224A	IHC 1:1600	Bethyl
CHD8	Rabbit	A301-225A	IHC 1:800 WB 1:1000	Bethyl
Doublecortin (DCX)	Rabbit	4604	WB 1:2000 IHC 1:500	Cell Signaling
GFAP	Mouse	KIT-0031	IHC 1:300	MAIXIN
GFAP	Rabbit	Z0334	WB 1:10000	Dako
NeuN	Mouse	MAB-0578	IHC 1:100	MAIXIN
NeuN	Mouse	Ab104224	WB 1:5000	Abcam
Olig2	Rabbit	ZA-0561	IHC 1:100	ZSGB
Olig2	Rabbit AB9610		WB 1:1000 Merck Millipore	
Homer	Mouse	Sc-17842	WB 1:2000	Santa Cruz
MBP	Rat	ab7349	WB 1:500	Abcam
Pax6	Rabbit	901301	IHC 1:500	Bio legend
TBR1	Rabbit	Ab31940	IHC 1:500	Abcam
PV	Rabbit	Ab11427	WB 1:1000	Abcam
Calbindin1	Rabbit	AB1778	IHC 1:500	Millipore
Tubulin	Mouse	T5168	WB 1:20000	Sigma
Iba1	Rabbit	019-19741	IHC 1:500	Wako
MYRF	Rabbit	ABN45	WB 1:1000	Merck Millipore
Sox9	Mouse	MABC277	WB 1:2000	Merck Millipore
Sox9	Rabbit	AB5535	IHC 1:500	Merck Millipore
Actin	Mouse	MAB1501	WB 1:5000	Merck Millipore
Vinculin	Rabbit	Ab129002	WB 1:10,000	Abcam

**Table:** 

Experimental models: cell lines		
HEK 293 cell	ATCC (cat# CRL-1573)	N/A

**Table:** 

Experimental models: organisms/strains		
CHD8 mutant monkeys		This paper

**Table:** 

Gene	sequence	length	experiment
*SRY*	TGCTTCTGCCATGTTAAGCGT CGGAAGCAAACTGCAACTGTTTTG	427 bp	Gender detection in T1, T2, T3
*CHD8*	TCAAGTTGGGATTGGATAAAG CACAATGACTCACTACAAACTTAT	110 bp	Deep amplicon sequencing
	TGTCCACGCAGACTTTAGTG GGCACAGACAGAACATGCCTT AGTGTGCTGACACCTGGTCTAGATA CTTGGAGCCTTCATCATCTTCCT	Nested PCR 641 bp	Genotyping in *CHD8* exon 19

**Table:** 

Recombinant plasmid		
Plasmid: Cas9-MLM3613 T7 promoter	Cas9 with CMV	Addgene Plasmid#4225
Plasmid: pSpCas9(BB)-2A-GFP used in cultured cells	Cas9 together with sgRNA	Addgene Plasmid#48138
Plasmid: sgRNA with T7 promoter	sgRNA with T7 promoter	Liangxue Lai’s lab

**Table:** 

Software and algorithms		
GraphPad Prism 8	Graphpad Software	https://www.graphpad.com
Stereo Investigator 5.4.3	Micro Bright Field Bioscience	www.mbfbioscience.com
Image J	National Institutes of Health	https://imagej.nih.gov/ij/docs
Aperio ImageScope	Leica	https://www.leicabiosystems.com/digital-pathology/manage/aperioimagescope/
MatLab (R2019b)	MathWorks	https://www.mathworks.com/products/matlab.html
SPM 12	WDCN	https://www.fil.ion.ucl.ac.uk/spm/software/spm12/
RadiAnt DICOM View	Medixant	https://www.radiantviewer.com/
Actical 3.1.1	Respironics	https://bmedical.com.au/productcategory-/activity-heatresearch/actical/
MicroView 2.5.0	Parallax Innovations	https://sourceforge.net/projects/microview/

**Table:** 

Other		
Axio Imager A2	Zeiss	Carl Zeiss, Germany
Zeiss LSM 800 Confocal Laser Scanning Microscope	Zeiss	Carl Zeiss, Germany
Leica Aperio GT45	Leica	Leica Biosystems, Germany
GE Locus SP Micro CT Scanner	GE	GE, USA
GE Discovery MR750 3.0 T Scanner	GE	GE, USA
Actical Physical Monitors	N/A	Respironics, USA
SONY FDR-AX45 Video Camera	SONY	SONY, Japan
JVC GC-P100BAC Video Camera	JVC	JVC, Japan
Infrared Video Camera C6p	HIKVISION	HIKVISION, China
Actical Physical Monitors	Respironics	Respironics, USA

### Monkey

*CHD8* mutant monkeys and age-, gender-matched control monkeys (*M. fascicularis*) were raised at Guangdong Landau Biotechnology Co. Ltd., which is an Association for Assessment and Accreditation of Laboratory Animal Care-accredited facility. Mutant and control monkeys used in this study were listed below. The commercial monkey diet (Ke-Ao, #HFZ-15kg, Beijing) was offered twice daily. Monkey health was monitored by veterinarians. All animal-related protocols were approved in advance by the Animal Care and Use Committee of Guangdong Landau Biotechnology Co. Ltd. This study was carried out in strict compliance with the “Guide for the Care and Use of Laboratory Animals of the Institute of Laboratory Animal Science (est. 2006)” and “the use of nonhuman primates in research of the Institute of Laboratory Animal Science (est. 2006)” to ensure the safety of personnel and animal welfare.

### Animal information

**Table:** 

Name	Gender		Genotype	Note	
M1	M		*CHD8* mutant	Aborted (E125)	
M2	M		*CHD8* mutant	Stillborn (E158)	
M3	M		*CHD8* mutant	Alive (54 M)	
T1, T2, T3	F, M, M		*CHD8* mutants	Aborted (E53)	
Control 1	F		Wild type	Cesarean (E130)	
Control 2	M		Wild type	Newborn (E160)	
Control 3	M		Wild type	Alive (54 M)	
Control 4	M		Wild type	Alive (54 M)	
Control 5	M		Wild type	Alive (54 M)	
Newborn-1	M		Wild type	Brain slice culture	
Newborn-2	M		Wild type	Brain slice culture	
Newborn-3	M		Wild type	Brain slice culture	
Newborn-4	M		Wild type	Brain slice culture	
Newborn-5	F		*CHD8* gRNA	Brain development analysis	
			injection at G55		
Newborn-6		M	*CHD8* gRNA injection at G55	Brain development analysis	
Newborn-7		M	Control gRNA injection at G55	Brain development analysis	

### Genotype analysis of targeted site

Genomic DNA was isolated from tissue lysate using the Quick-gDNATM MiniPrep (Zymo Research) according to the manufacturer’s protocol. The target sites were PCR amplified with primers listed above. The PCR products were purified with Qiaquick PCR Purification Kit (Qiagen, 28106). For T7E1 cleavage assay, purified PCR products were denatured and reannealed in NEBuffer 2 (NEB) using a thermocycler. Reannealed PCR products were digested with T7 endonuclease 1 (NEB, M0302L) and separated by 2.5% agarose gel. The PCR products were sub-cloned into the pEASY-blunt simple cloning vector (Transgene) and transformed to competent DH5α cells. After overnight culture at 37 °C, emerged colonies were picked up randomly and sequenced. Deep amplicon sequencing was performed on various tissues to analyze mosaic in mutant monkeys.

### RNA preparation and sequencing

Total RNA was extracted from the prefrontal cortex (PFC) of *CHD8* mutant M2 and age-matched control-2 (three samples each). Only samples with RNA integrity number (RIN) over 6.8 were used for cDNA library construction. Sequencing was performed on a single lane of an Illumina HiSeq 4000 to produce 150 bp paired-end reads. We performed three independent replicates from adjacent areas for each animal. The clean reads were aligned with Bowtie2^61^ to the cynomolgus monkey genome (version 5.0). Duplicates were removed using Sambamba (version 0.6.8). Unique mapped reads were assigned to each gene according to Ensembl genome annotation. Differential expression analysis was performed by DEseq2.

### Off-target analysis by whole-genome sequencing

Whole-genome sequencing data was used to analyze off-targets in CRISPR/Cas9 editing. Extracted DNA was sequenced as paired-end reads on the Illumina HiSeq2000 platform. Possible off-target sites of sgRNA with no more than five mismatched sites were identified using the bio-information-based search tool, Cas-OFFinder. A total of 1326 possible off-target regions with NAG or NGG PAM sequences were identified. Relative sequencing depth for most likely off-target loci by sgRNA in M3 was calculated by normalizing the number of mapped reads in those loci to the genome-wide average of mapped reads. We found no off-target mutations in mutant monkeys M1 and M2. A mutation site with 5 bp mismatches from *CHD8* gRNA was found in M3, which is likely an inherited or de novo mutation.

### Organotypic monkey brain slice culture and lentiviral infection

The brain cortex of newborn male monkeys at postnatal day 1–3 was carefully dissected on ice, directly transferred into ice-cold artificial cerebrospinal fluid (ACSF) (SL6630, Coolaber, Beijing, China) equilibrated with carbogen (95% O_2_, 5% CO_2_). The tissues were cut to generate brain slices at 300 μm thickness using a Leica VT1200S vibrating blade microtome (Leica, Wetzlar, Germany). The slices were then transferred onto 30 mm Millicell membrane inserts (0.4 μm; Millipore, Bedford, MA, USA) and kept in six-well cell culture plate containing 1.5 mL media (Neurobasal/DMEM 1:1, 5% fetal bovine serum, 5% horse serum, 1% N2, 2% B27, 2 mM lGlutamax, 5 ng/mL GDNF, 100 U/mL Pen-Strep; all from Gibco). The plates were incubated at 37 °C in a 5% CO_2_ humidified incubator. The medium was replaced with fresh medium three times each week. After 6 h of culture, virus infection was carried out according to experimental needs. The viral vectors used were lentiviral-CHD8 gRNA/CMV-GFP and lentiviral-EFs Cas9. These viral vectors had been packaged by Guangzhou IGE Biotechnology LTD to generate purified viruses. The genomic titer of purified viruses was ~10^9^ vg/mL determined by PCR method.

We generated lentiviral vectors to express CHD8 gRNA or control gRNA that did not target any gene^62^ (Supplementary Fig. S9a). To examine the effect of knocking down *CHD8* on glial proliferation, cultured monkey brain slices were infected with lentiviral viruses (each brain slice infected with 1 µL lentiviral gRNA and lentiviral Cas9). These viruses were diluted in culture medium and then added to the brain cortical slices containing the LV area. After 10–14 days of viral infection, immunofluorescent staining was performed to examine glial cell numbers and morphology. We used four newborn monkeys for organotypic brain slice culture and found 3 of them provided brain slices with effective lentiviral infection.

### In utero delivery of lentiviruses to the fetal monkey brain

The pregnancy after timed mating was assessed periodically by ultrasound examination, and pregnancy was confirmed with ultrasound evidence of fetal heart activity. Pregnant monkeys having fetal monkeys at embryonic day 55 (E55) or gestational day 55 (G55) were selected for in utero injection. After induction of anesthesia of the pregnant monkey with intramuscular injection of Zoletil^®^50 at a dose of 5 mg/kg, a 25-G Quincke needle (BectonDickenson) was used to target the LV of the fetal monkey brain closest to the anterior maternal abdomen under continuous ultrasound imaging. Lentiviruses (50 μL lentivirus *CHD8* or control gRNA mixed with 100 μL lentiviral Cas9 were injected as a slow bolus to deliver a total dose of 1.0 × 10^8^ vg (lentiviral gRNA) and 2.0 × 10^8^ vg (lentiviral Cas9) in each fetus. The correct delivery would result in ventricular swelling, and the needle was removed immediately. The injected fetus was monitored for another 15 min. We obtained two male and one female newborn monkeys, which were delivered naturally. These newborn monkeys were euthanized with intramuscular injection of Barbiturates with the dose of 100 mg/kg at postnatal day 7 or 8 to isolate their brains for analysis.

### Western blotting analysis and immunohistography

For western blotting analysis, PFC or other brain region was homogenized in RIPA buffer (Hua Xing Bo Chuang, with 1× protease inhibitor cocktail) on ice. Protein lysates were separated by SDS-PAGE and transferred onto PVDF membranes (Millipore). The primary antibodies used are listed in Materials and methods. Specific bands were quantified by Image J and normalized to α-tubulin or other reference protein expression.

For immunohistography, the brains of controls and mutants were dissected out and fixed for 48 h in 4% paraformaldehyde. Different brain regions including PFC and cerebellum were paraffin-embedded. Paraffin-embedded tissues were sliced into 4-μm-thick sections. HRP-conjugated secondary antibodies (anti-mouse or anti-rabbit, 1:1000, Dako) were used. DAB (3, 3'-diaminobenzidine) staining was used for chemiluminescent detection. Hematoxylin staining was performed to label the nucleus. Images were acquired with a Leica Aperio GT450 slide scanner. For cell density analysis, cells within the certain area were counted manually. LFB staining (RY-0013, Zhong Ke Wan Bang) was performed to analyze myelin changes in mutant monkeys.

### MRI image acquisition

Animals were anesthetized by ketamine (10 mg/kg, i.m.) and placed into the scanner in the prone position for T1-weighted 3-dimensional MR scan. The scans were collected on a 3.0 T MR machine (GE 750) using a custom-designed 8-channel radio-frequency surface head coil at Jinan University. We performed T1 sequence, with the following parameters: TR = 9.5 ms, TE = 4.0 ms, slice thickness = 0.5 mm, slice per slab = 196, matrix size = 256 × 256 mm, FOV = 150 × 150 mm, and voxel size = 0.5 × 0.5 × 0.5 mm.

### Regional volumetric computation

The statistical parameter mapping software (SPM 12, Wellcome Department of Cognitive Neurology, London, UK) with in-house MatLab scripts was employed to perform voxel-based morphometry (VBM)^63^ for calculating the volume of brain regions of each animal. A study-specific anatomical template was built and tissue classification was performed using the Diffeomorphic Anatomical Registration Through Exponentiated Lie algebra (DARTEL) pipeline^64^. First, the T1-weighted images were registered to the rhesus monkey brain template^64^, resulting in individual T1-weighted images in the standard space with an isotropic spatial resolution of 0.3 mm. The unified segmentation method^65^ was then used to segment these images into gray matter (GM), white matter (WM) and cerebrospinal fluid (CSF). The tissue segmentations were used to generate the final study-specific template, GM, WM, and CSF probability maps using the DARTEL pipeline. The individual GM, WM, and CSF segmentation were then spatially normalized to the study-specific template. Modulated tissue maps were generated by using the Jacobian determinant of the deformation field obtained in the normalization process to encode the expansion or contraction of voxels. Therefore, the tissue volume in each voxel was preserved in the modulated images, and the tissue volume of a given region of interest (ROI) could be estimated as the summation of the modulated voxel intensities multiplied by the voxel size. The modulated tissue maps were spatially smoothed for noise reduction using a Gaussian kernel with full width at half maximum of 0.9 mm. The brain atlas^66^ was affine registered to the study-specific template generated with the DARTEL pipeline, and the ROIs defined by the regional boundaries in the atlas were used for brain region volume calculation. The volumes of gray matter and white matter in each ROI were calculated separately.

### Quantification and statistical analysis

#### Analysis of RNA-seq data

Differentially expressed genes were identified using the following thresholds: log_2_-fold change > 1.5 and Benjamini–Hochberg adjusted *P* value < 0.05. For GO enrichment analysis, enriched terms were defined using fisher’s exact test with the following filters: odds ratio > 2 and Benjamini–Hochberg adjusted *P* value < 0.05.

#### Analysis of immunohistochemical data

For quantification of Olig2-positive cells in immunostaining of the brains of *CHD8* mutant monkeys, rectangles of equal size (such as 0.04 mm^2^) per picture (×20) were quantified for positively stained cells. Positive nuclei in the assigned areas were counted manually and normalized for the area surface or total cell number. For quantification of GFAP staining intensities in control vs. mutant, the total intensity was measured using Image J software. Brightness and contrast were adjusted where needed.

For quantification of glial cells in newborn monkeys that were injected with lentiviral CHD8 gRNA and Cas9 at E55, we selected GFP-positive brain regions for quantification because lentiviral gRNA also expressed GFP so that GFP-positive areas reflect the expression of lentiviral gRNA and Cas9 when both were mixed for injection. Each brain was sectioned serially using a sliding microtome at 40 μm. Sections were collected into tissue collection solution and stored at −20 °C until processing. Brain sections were first examined under a fluorescent microscope to examine GFP signaling in order to ensure that the brain region had been transduced by lentiviral vectors. This process is necessary to obtain the viral transduced brain region, as lentiviral transduction in the fetal brain was restricted to some extent. We used anti-Sox9 to identify astrocytes and anti-olig2 to identify oligodendrocytes. Because these antibodies label glial nuclei so that glial cells could be unequivocally assessed for their numbers and density. In addition, DAPI staining was used to confirm the presence of nuclei of glial cells. To ensure antibody specificity, staining was also performed without the primary antibody but secondary antibody. Images containing Sox9 or olig2-positive cells at ×200 magnification or 0.31 mm^2^ were acquired by Zeiss (Carl Zeiss, Germany) and processed with Zeiss software (Carl Zeiss, Germany). Every 32nd section (or 1.3 mm apart) was used for quantitative analysis of immunostaining, and six and eight sections from the brain cortical region in control gRNA/Cas9 and *CHD8* gRNA/Cas9, respectively, targeted monkeys were used to quantify the numbers of cells. At least four images (20×) from each brain section were taken to obtain the average numbers of glial cells per mm^2^. The staining results were scored by two investigators blinded to the genotypes (control and CHD8 targeted) of the monkey brains.

A similar method was also used to quantify glial cells in the monkey brain slices that were infected by lentiviral CHD8 or control gRNA with lentiviral Cas9. Because each monkey brain can provide multiple brain slices (400 µm each slice), at least three slices from each animal were infected by lentiviral CHD8 or control gRNA with lentiviral Cas9. After infection for 14 days, the infected brain slices were fixed and sectioned at 40 µm. Because each brain slice was infected by lentivirus and cultured independently, the number of brain slices represents biological replicates. We used a total nine brain slices from three newborn monkeys (three slices per animal) for quantitative analysis. At least three brain sections (40 µm) containing GFP-positive areas, which was identified under a fluorescent microscope, in each brain slice were used for quantification of astrocytes labeled by anti-Sox9 and oligodendrocytes labeled by anti-olig2. The infected brain regions next to the LV were selected for quantification. At least four images (×20) in each brain section were taken for counting the number of glial cells to provide the average number of glial cells in each brain slice that was used for quantification. The average numbers of glial cells per mm^2^ were used for comparison between control and CHD8-targeted monkey brain slices.

### Statistical analysis

Data analysis was conducted using GraphPad Prism version 6 or 8. The normality of the data was analyzed first. Data were analyzed using two-tailed Student’s *t* test or Mann–Whitney *U* test for comparing two groups and univariate or two-way analysis of variance (ANOVA) and subsequent post hoc *t* tests using a Dunnett. All data were presented as means ± SE. The alpha level was set at *P* = 0.05 (NA, not significant, *P < 0.05; **P < 0.01; ***P < 0.001).

## Supplementary information

Supplementary figures

## Data Availability

All data generated or analyzed during this study are included in this article and its supplementary information files. The sequence data of RNA-seq and whole-genome sequencing of monkeys reported in this project have been deposited to Genome Sequence Archive (GSA, https://ngdc.cncb.ac.cn/gsa/) with accession number CRA003937.
