# Supplementary material for: CHD8 mutations increase gliogenesis to enlarge brain size in the nonhuman primate
Source: Cell Discov. 2023 Mar 7;9:27. doi: 10.1038/s41421-023-00525-3 (PMC9988832; doi:10.1038/s41421-023-00525-3)
Supplement: Supplementary file 1 — Supplementary figures [file 41421_2023_525_MOESM1_ESM.pdf]

Supplementary information

CHD8 mutations increase gliogenesis to enlarge brain size in the non-human primate

Bang Li[1*], Hui Zhao[2*], Zhuchi Tu[1*], Weili Yang[1*], Rui Han[1], Lu Wang[3], Xiaopeng Luo[1], Mingtian Pan[1], Xiusheng Chen[1], Jiawei Zhang[1], Huijuan Xu[2], Xiangyu Guo[1], Sen Yan[1], Peng Yin[1], Zhiguang Zhao[2], Jianrong Liu[4], Yafeng Luo[4], Yuefeng Li[5], Zhengyi Yang[6], Baogui Zhang[6], Zhiqiang Tan[3], Hao Xu[3], Tianzi Jiang[6], Yonghui Jiang[7], Shihua Li[1], Yong Q. Zhang [2#], Xiao-Jiang Li[1#]

This PDF file includes

Supplementary Fig. S1-S13

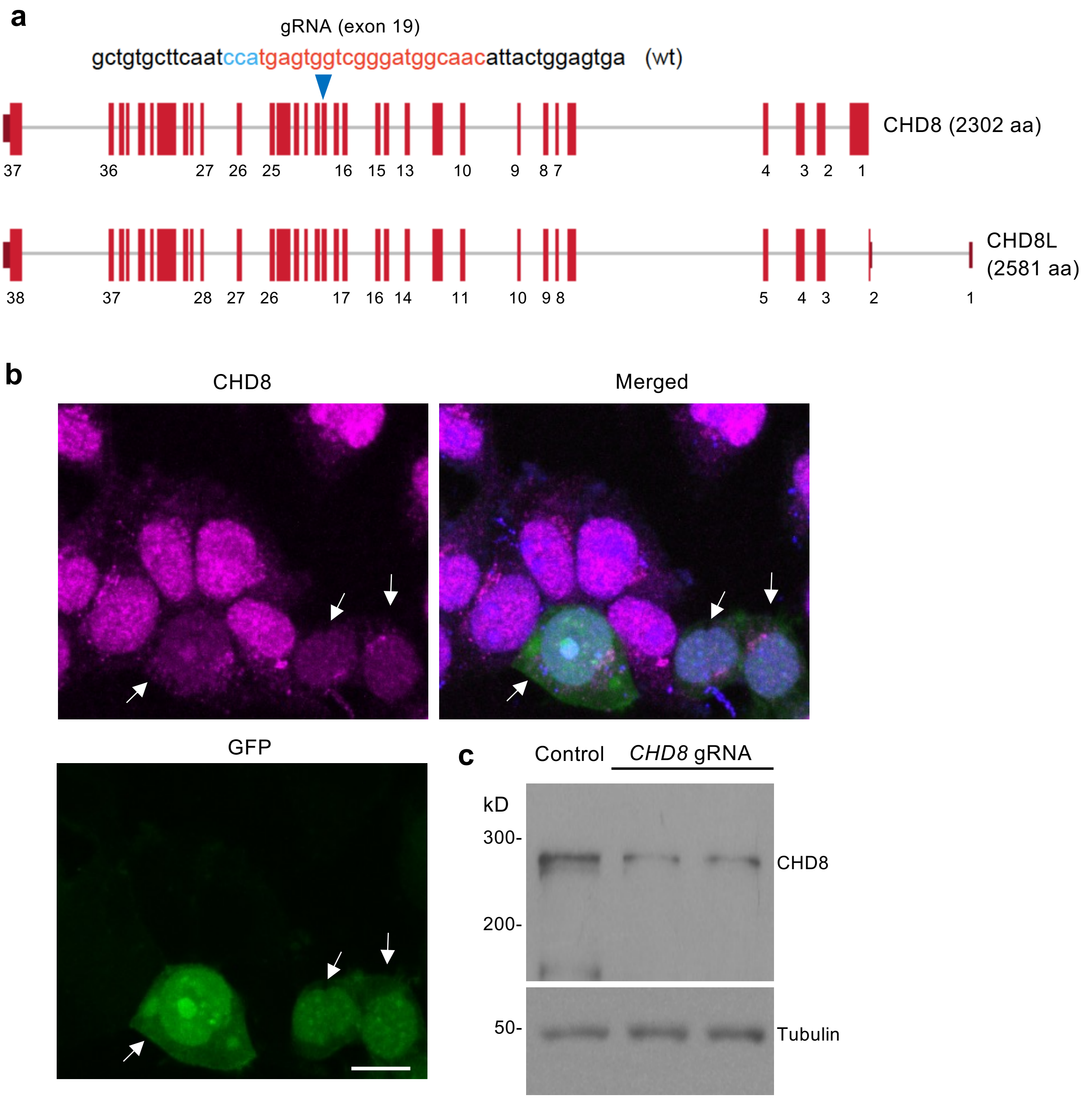

Supplementary Fig. S1. Targeting the primate *CHD8* and reduced CHD8 expression in cultured cells

(**a**) Genomic DNA structure of the human *CHD8* (NM_001170629.1 and NM_020920.3). Exon 19 is targeted by *CHD8* gRNA.

(**b**) A plasmid expressing both *CHD8* gRNA and GFP was co-transfected with Cas9 into HEK 293 cells. GFP-positive cells (arrows) show the reduced immunostaining by anti-CHD8. Scale bar: 20 μm.

(**c**) Western blotting of transfected cells showing the reduced level of CHD8 after transfection with *CHD8* gRNA and Cas9.

a

Deep sequencing of targeting site in triplet embryos

	tissue	wt reads	-2/+1 bp reads	-4 bp reads	mutation rate
T1	embryo	1254	42	26	5.1%
T2	embryo	1798	637	46	27.5%
T3	embryo	908	193	22	19.1%

b

TA clone sequencing of M1 targeting site

	brain	heart	liver	lung	spleen	gut	kidney	skin	bone	muscle	testis	umbilical cord
wt	0	1	0	2	0	0	0	3	0	3	0	0
-4/+4	10	7	9	1	7	8	13	9	11	3	13	7
-4/+2	10	11	11	9	13	10	4	7	6	5	5	7
Mutation rate %	100	94.7	100	83.3	100	100	100	84.2	100	72.7	100	100

TA clone sequencing of M2 targeting site

	brain	heart	liver	lung	spleen	gut	kidney	skin	bone	muscle	testis	umbilical cord
wt	11	5	12	9	10	8	3	9	7	11	9	7
+16	17	14	7	7	8	8	8	8	8	5	7	11
Mutation rate %	60.7	73.7	36.8	43.8	44.4	50	72.7	47	53.3	31.3	43.8	61.1

Supplementary Fig. S2. Analysis of *CHD8* mosaic mutations in monkey offspring

(**a**) Deep sequencing of genomic DNAs from fetal tissues of aborted triplets (T1, T2, and T3) showing mutation rates of the *CHD8* gene.

(**b**) The targeted *CHD8* DNAs from different tissues in *CHD8* mutant monkeys (M1 and M2) were amplified by PCR and subcloned for sequencing, revealing different targeting rates in different tissues due to the mosaic nature of CRISPR/Cas9 targeting. wt: wild type *CHD8* DNA sequences. -4/+4, -4/+2, and +16 are mutation types. The numbers in the table are sequenced clones carrying wt or mutant DNAs.

a

sgRNA 5'-GTTGCCATCCCGACCACTCATGG-3'

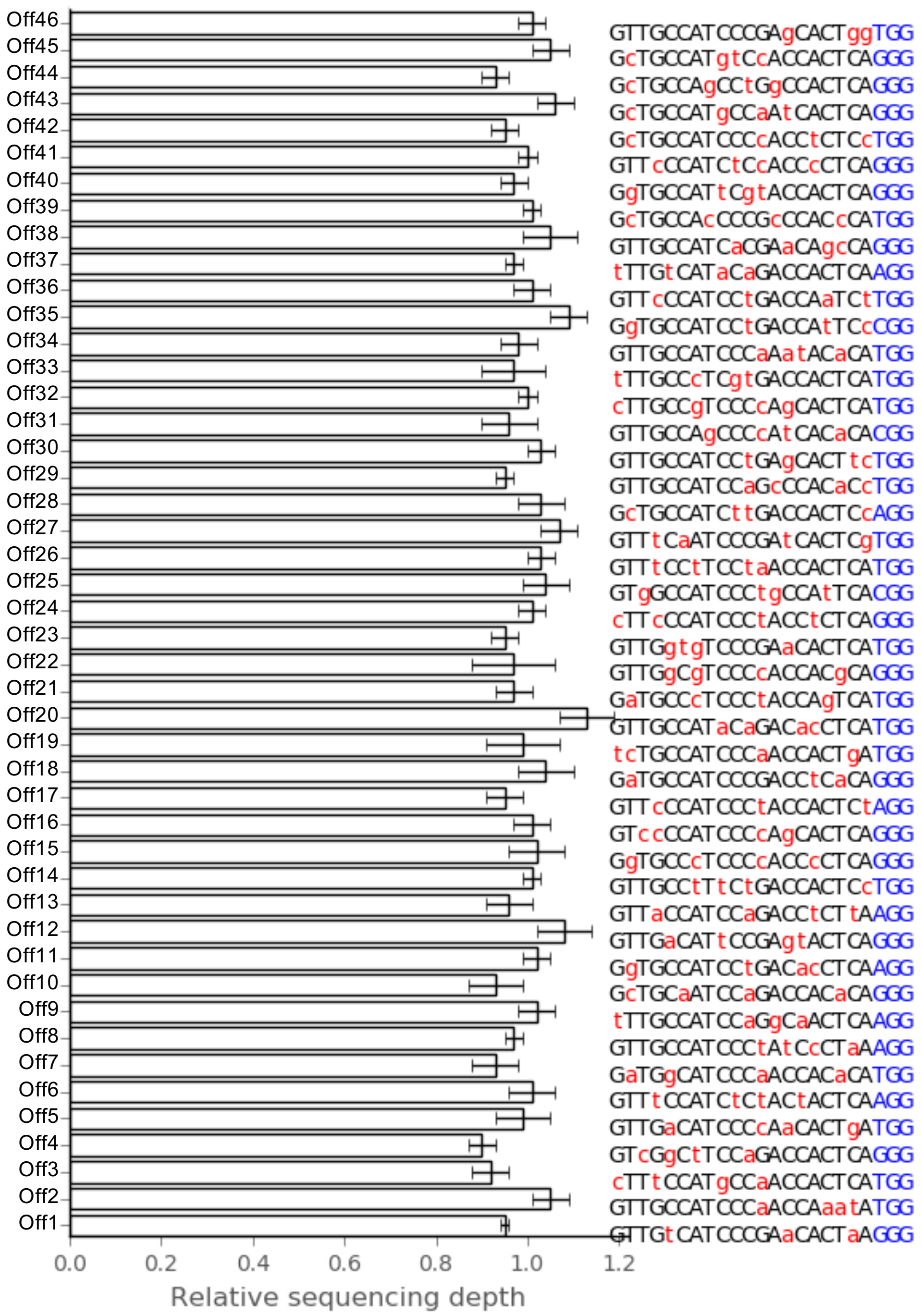

b

Numbers of off-targets

Number mismatches	PAM (NGG+NAG)	M1 (brain cortex)	M2 (brain cortex)	M3 (blood)
1	0	0	0	0
2	0	0	0	0
3	11	0	0	0
4	106	0	0	0
5	1209	0	0	1

Supplementary Fig. S3. Off-target assays of *CHD8* targeting

(**a**) Whole genome sequencing of the blood sample of M3 shows no obvious off-targeting events.

(**b**) Summary of whole genome sequencing of the genomic DNAs from the brain cortex of M1 and M2 and the blood of M3. The numbers in M1, M2, and M3 represent targeting events in the potential targeting sequences with PAM and different mismatched numbers (1-5) of nucleotides.

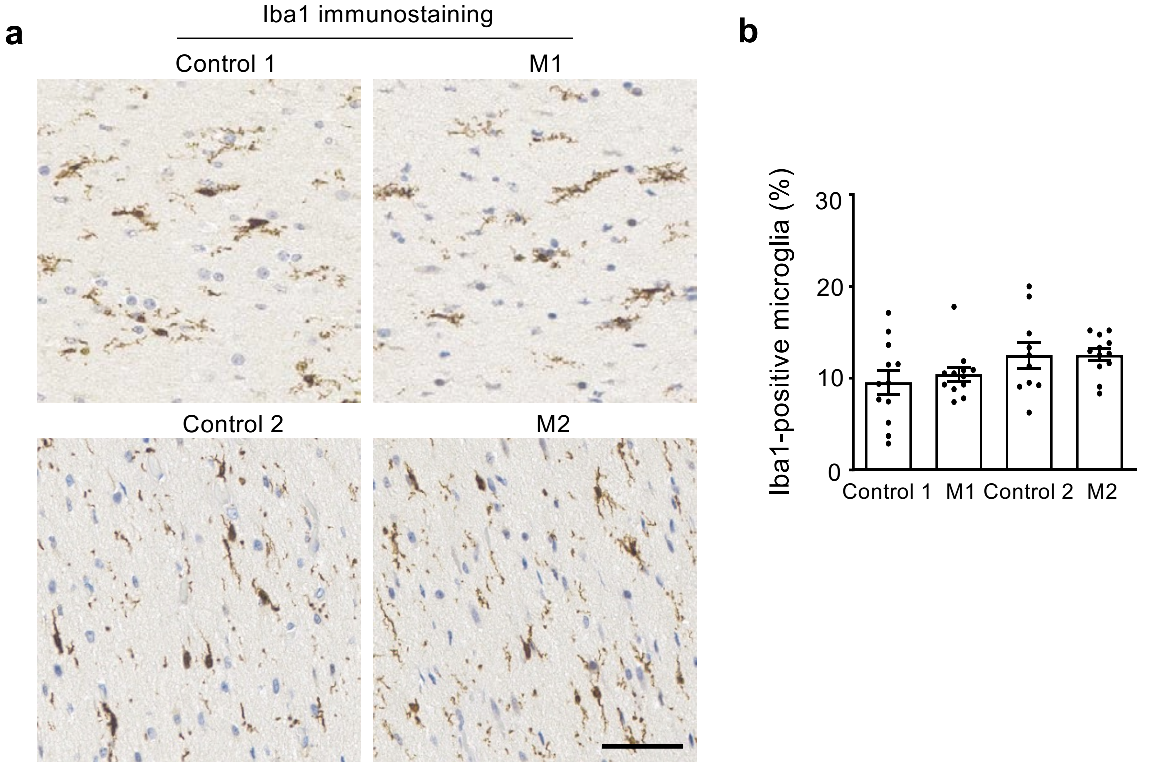

Supplementary Fig. S4. No influence of reduced CHD8 on microglial cells in the cortex

(**a**) Immunostaining of the white matter with antibody to Iba1, a microglial marker protein, showing no difference between control-2 and M2. Scale bar: 50 µm.

(**b**) The percentage (mean+/-SE) of microglial cells is presented.

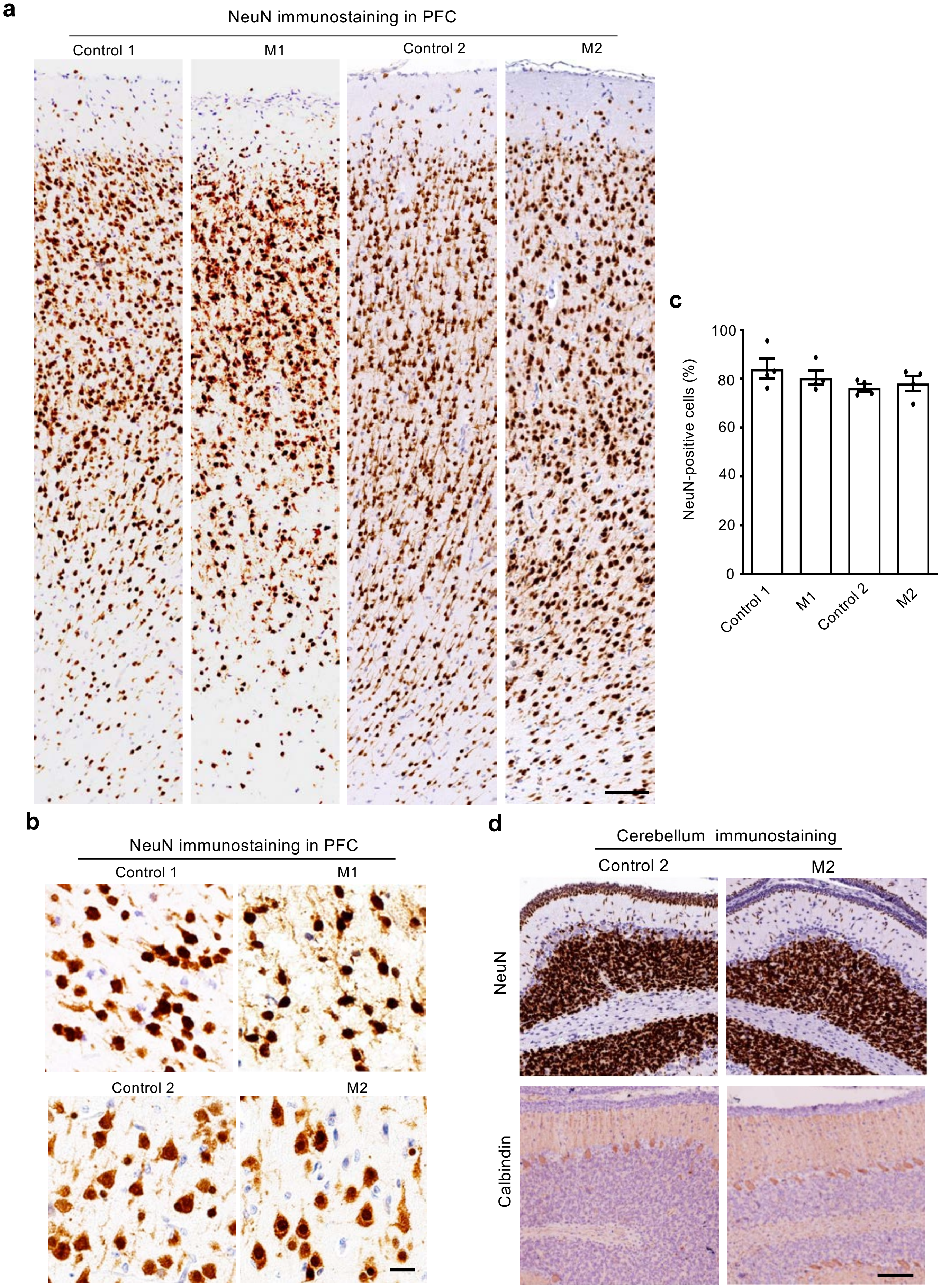

Supplementary Fig. S5. Neuronal cells in the prefrontal cortex in control and *CHD8* mutant monkeys (a) Immunostaining of the white matter and all layers of prefrontal cortex with an antibody to NeuN, a marker protein for neuronal cells. Scale bar: 100 μm.

(**b**) Enlarged images of the cortex stained by anti-NeuN. Scale bar: 20 μm.

(**c**) Density of NeuN-positive neurons in the cortex of control and *CHD8* mutant monkeys. The data were obtained by counting 4 images per group and presented as mean+/-SE.

(**d**) Immunostaining of the cerebellum with antibodies to NeuN (upper) and calbindin (lower) showing no obvious difference between control-2 and M2. Scale bar: 100 μm.

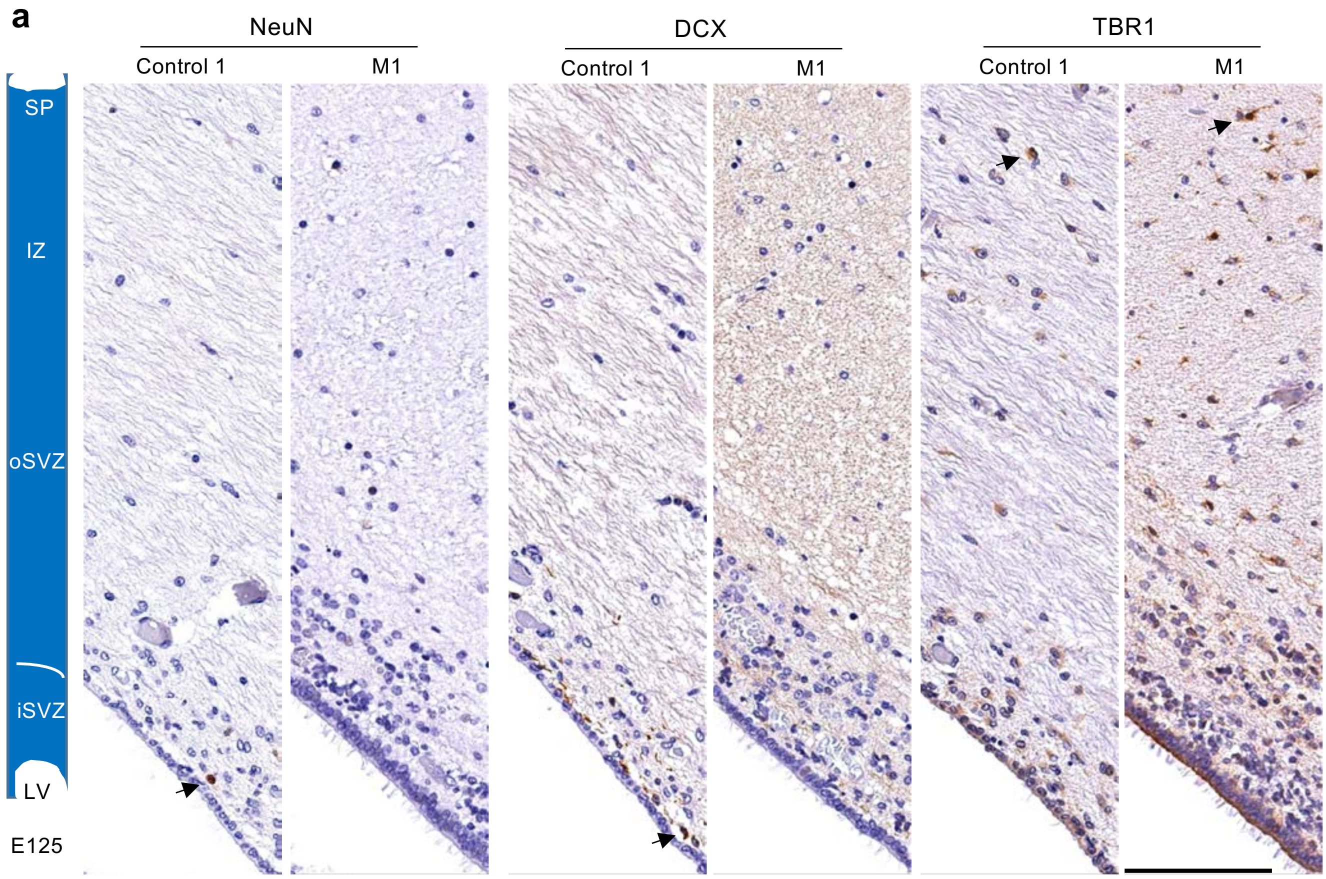

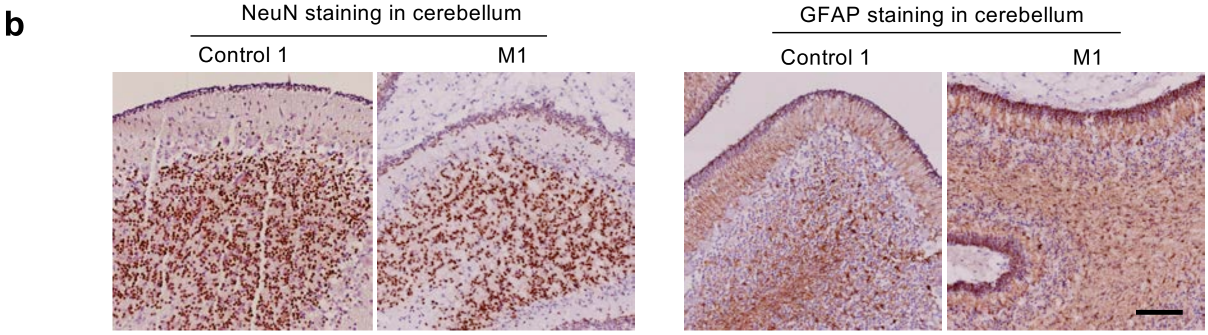

Supplementary Fig. S6. No alteration in neurogenesis in M1 monkey brain

(**a**) Immunostaining of the brain slice containing the inner subventricular zone (iSVZ), outer subventricular zone (oSVZ), the intermediate zone (IZ), and subplate (SP) with neuronal markers NeuN (mature neurons), DCX (immature neurons) and TBR1 (migrating neurons) in control-1 and M1 at E125-E130. Black arrows indicate few positive cells in each staining. Scale bar: 100 µm.

(**b**) Immunostaining of the cerebellum with antibodies to NeuN and GFAP did not show obvious difference between control-1 and M1. Scale bar: 100 µm.

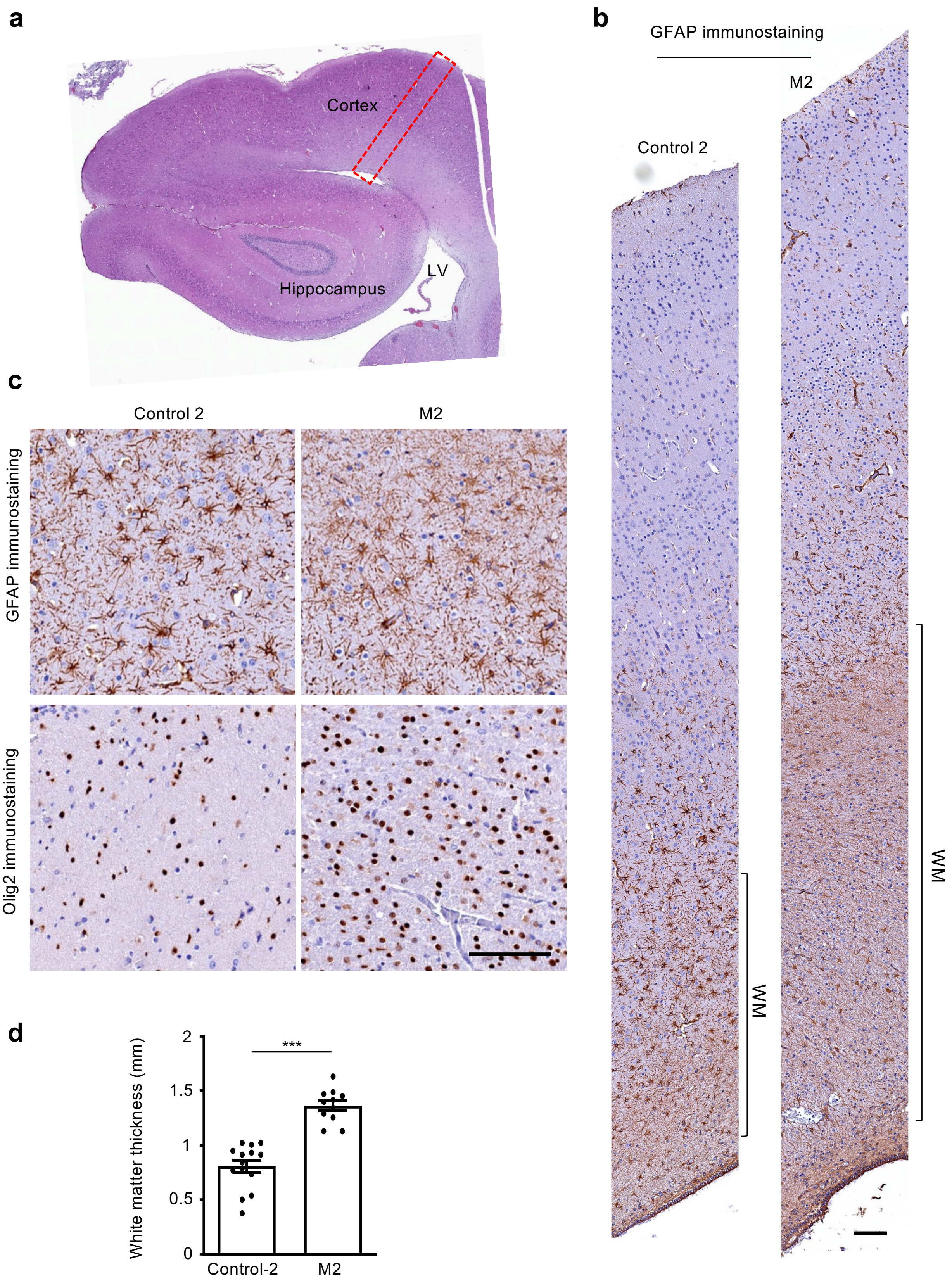

Supplementary Fig. S7. Increased glia density in the white matter of M2 monkey temporal lobe

(**a**) Hematoxylin-eosin staining of brain tissue with red rectangular for cutting brain slices.

(**b**) Low magnification micrographs of the anti-GFAP-labeled brain cortical region of control 2 and M2 monkeys. WM: white matter. Scale bar: 100 μm.

(**c**) GFAP and oligo2 immunostaining of the brain white matter region of control 2 and M2 monkeys. Scale bar: 100 μm.

(**d**) Quantitation of the thickness of white matter in the control 2 and M2 monkey temporal lobe. *** p<0. 001 in student's t test.

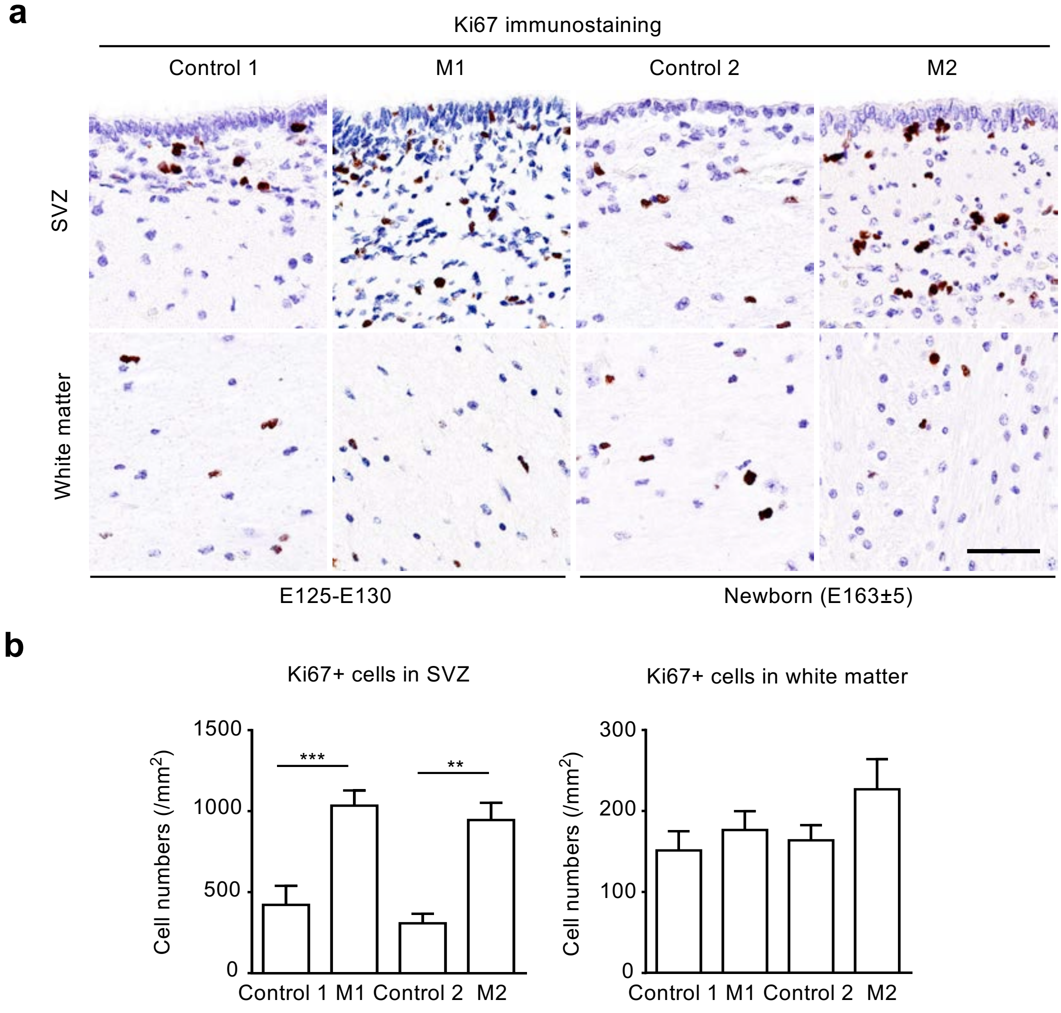

Supplementary Fig. S8. Immunostaining of proliferative marker Ki67 in M1, M2 and control monkeys

(**a**) Ki67 immunostaining of the subventricular zone(SVZ) and white matter in M1, M2, and their controls. Scale bar: 50 µm.

(**b**) Quantitation of the Ki67-positive cells (mean± SE) in SVZ and white matter. The data were obtained by counting 6-8 images from each animal. Statistical analysis by One way ANOVA and Tukey's multiple comparisons test. ** p<0. 01; *** p<0. 001.

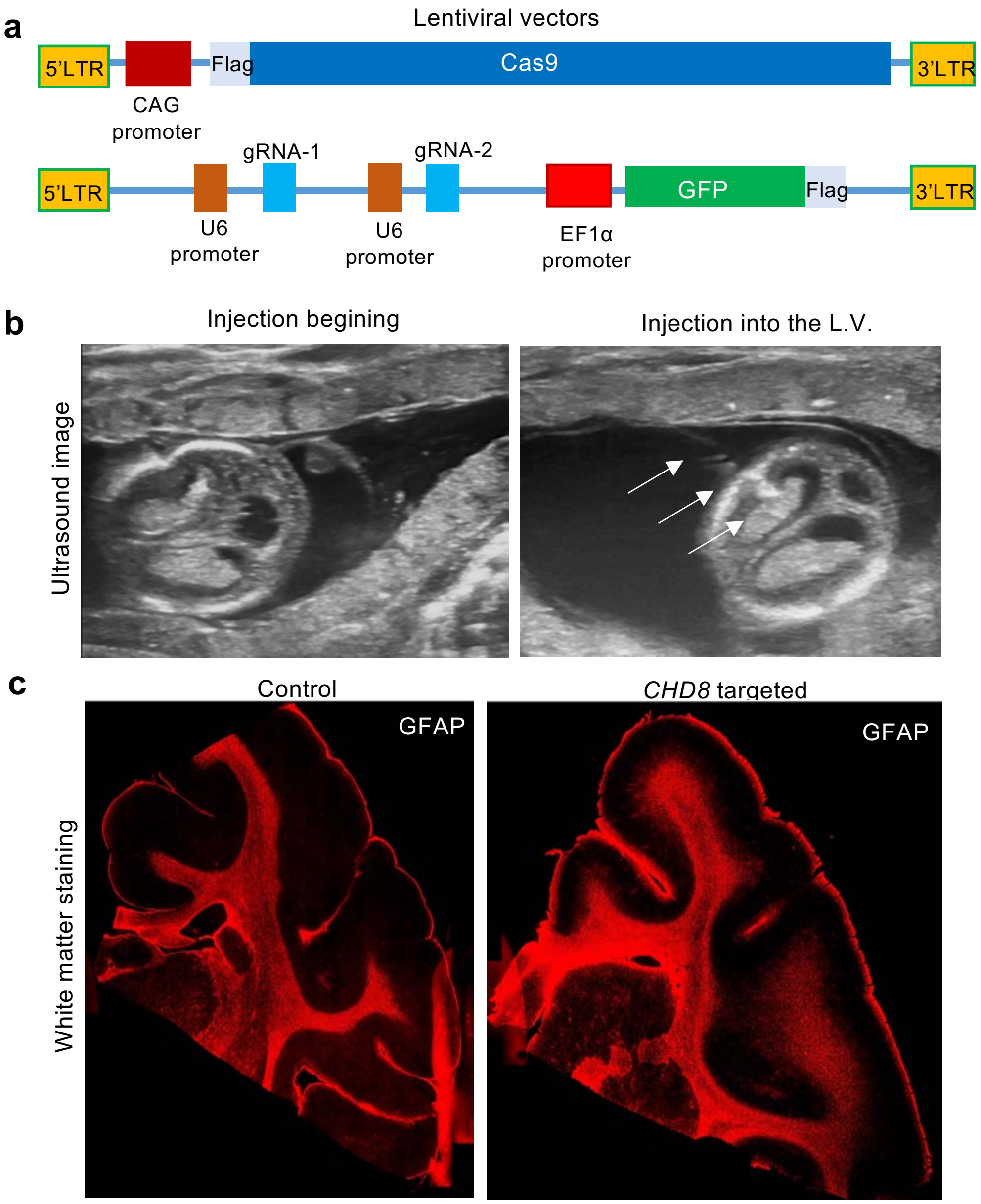

Supplementary Fig. S9. Fetal brain injection of lentiviral vectors led to transgene expression in neuronal and glial cells.

(**a**) Lentiviral vectors used to deliver Cas9, *CHD8* or scrambled gRNA and GFP.

(**b**) Ultrasound images showing injection beginning and into the lateral ventricle (LV) in a fetal monkey brain at G55.

(**c**) Immunostaining of the newborn monkey brain with anti-GFAP clearly identified white matter in control and CHD8 targeted monkey brains.

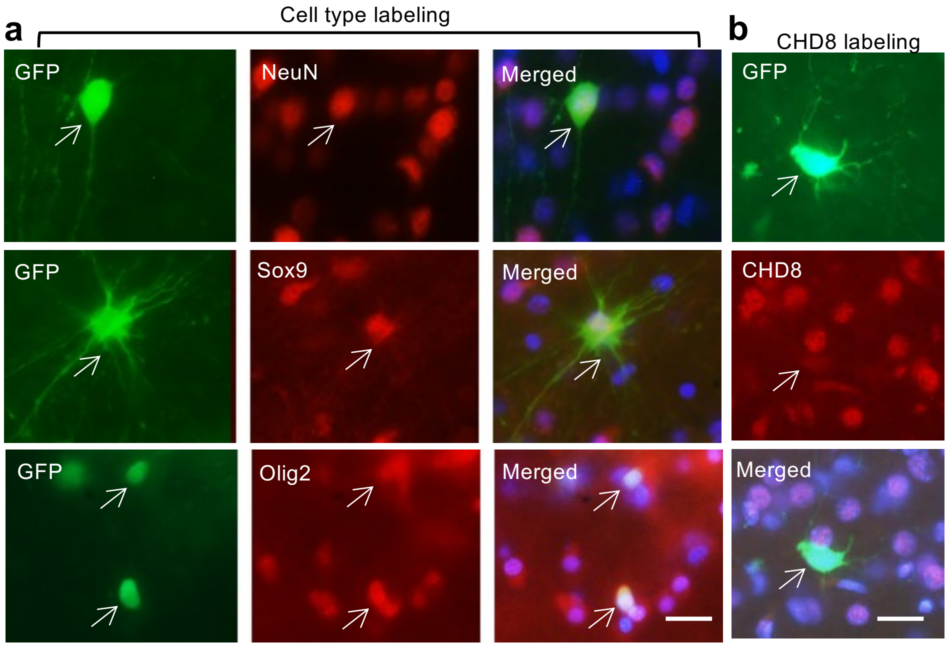

Supplementary Fig. S10. Fetal brain injection of lentiviral vectors led to transgene expression in neuronal and glial cells.
(**a**) Double immunostaining of the brain cortex in newborn monkey showing the expression of GFP, which reflects lentivirus-mediated transgene expression, in neuronal (NeuN) and glial (Sox9 and Olig2) cells. Scale bar: 10 μm.
(**b**) Targeting *CHD8* by lentiviral infection (GFP) reduced the expression of CHD8 in the infected cells (arrow). Scale bar: 10 μm. Blue in (A) and (B) is DAPI staining of the nucleus.

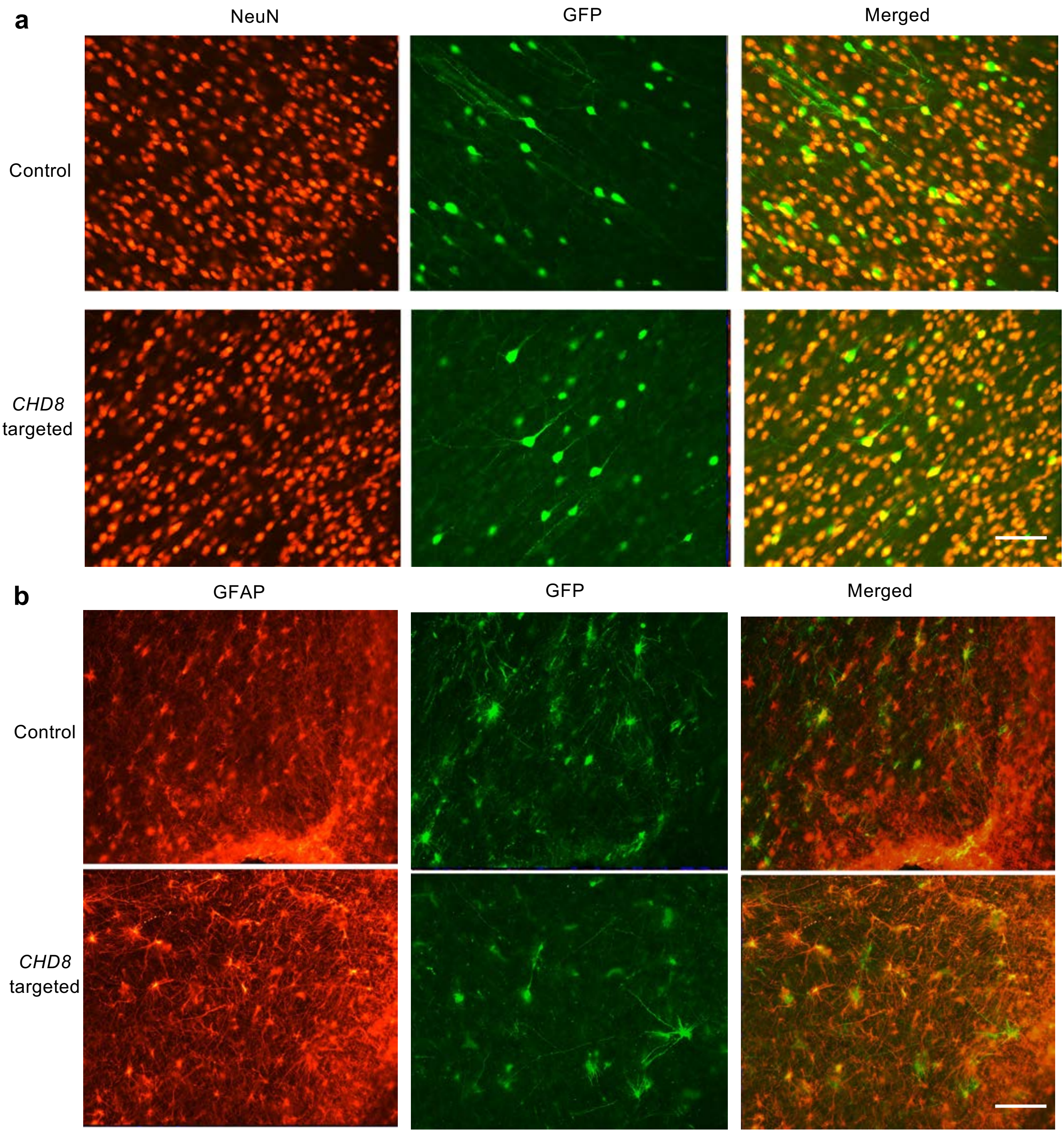

Supplementary Fig. S11. Comparison of neuronal and glial cells in the control and CHD8 targeted monkey brain.
The brain sections from newborn monkeys (control and CHD8 targeted) were immunostained by antibodies to NeuN (**a**) and GFAP (**b**). Scale bars: 25 μm.

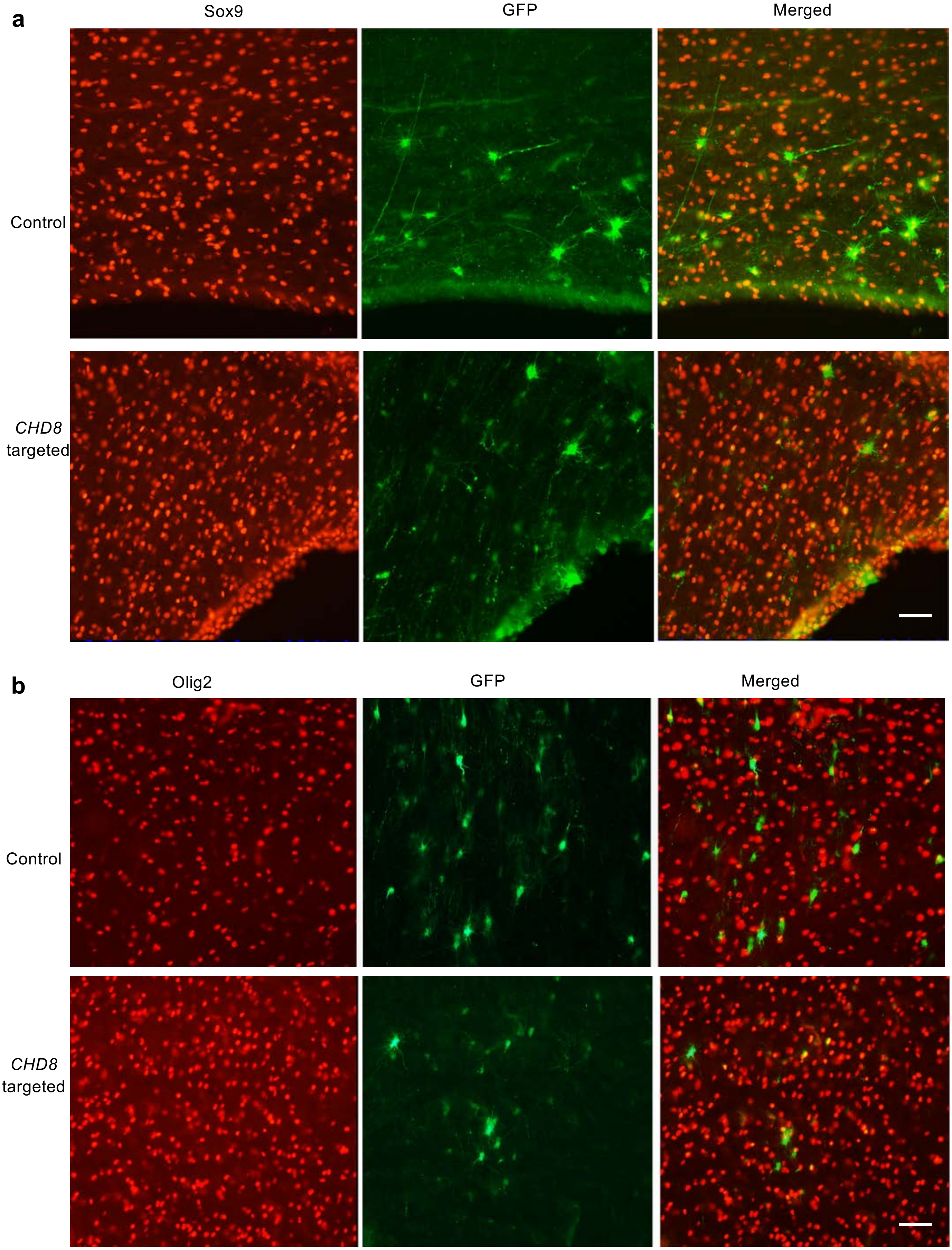

Supplementary Fig. S12. Comparison of astrocytes and oligodendrocytes in the control and CHD8 targeted monkey brain.
The brain sections from newborn monkeys (control and CHD8 targeted) were immunostained by anti-Sox9 for labeling astrocytes (**a**) and anti-olig2 for labeling oligodendrocytes (**b**). Scale bars: 25 μm.

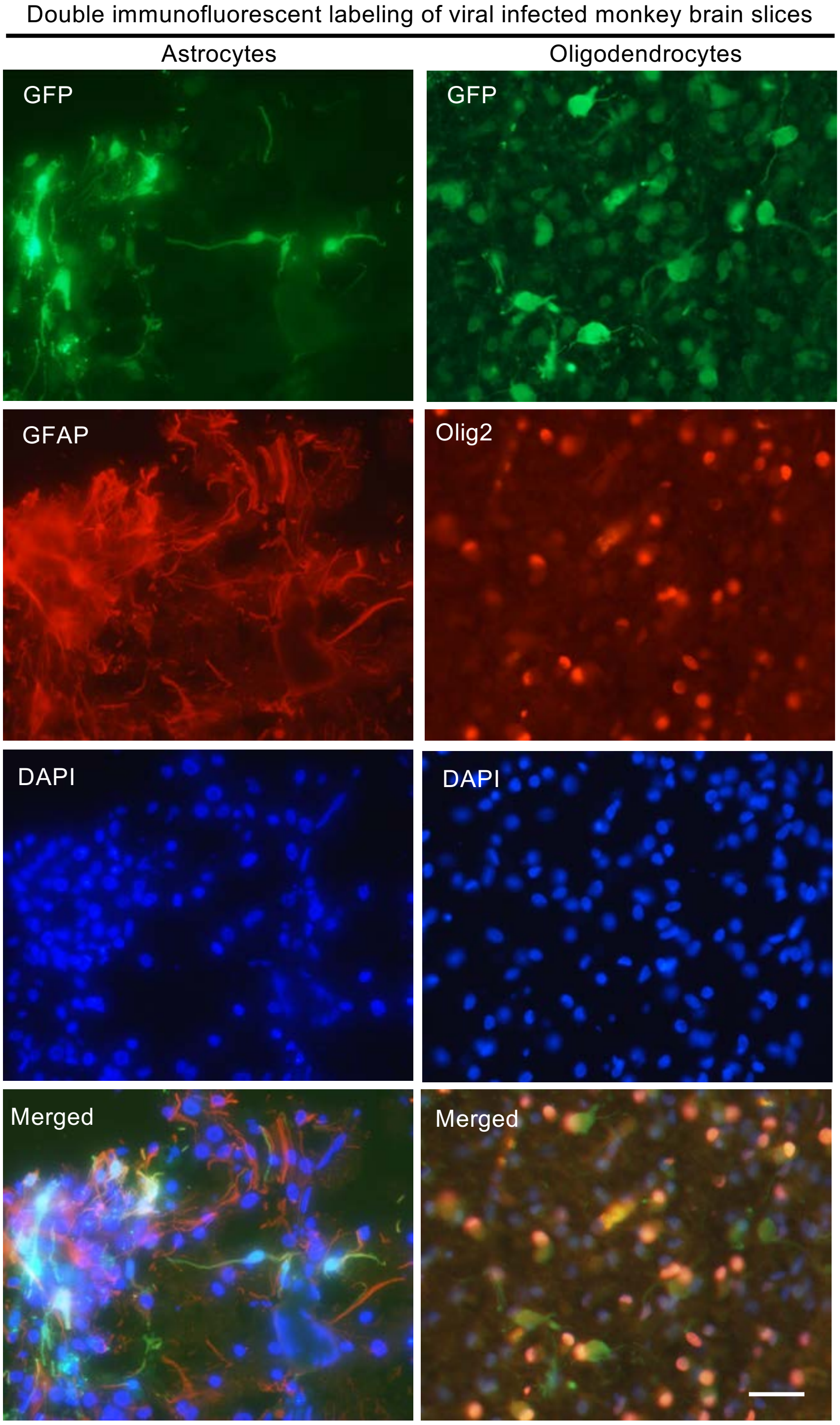

Supplementary Fig. S13. Lentiviral infection of brain organoid slices from newborn monkeys.

Double immunostaining cultured brain slices that were infected by lentiviral *CHD8* gRNA/Cas9 for 10 days. Glial cells labeled by anti-GFAP (astrocytes) and anti-olig2 (oligodendrocytes) were transduced by lentiviral vectors. Scale bar: 10 μm.
